# Reinforcement Learning Disruptions in Individuals With Depression and Sensitivity to Symptom Change Following Cognitive Behavioral Therapy

**DOI:** 10.1001/jamapsychiatry.2021.1844

**Published:** 2021-07-28

**Authors:** Vanessa M. Brown, Lusha Zhu, Alec Solway, John M. Wang, Katherine L. McCurry, Brooks King-Casas, Pearl H. Chiu

**Affiliations:** 1 Department of Psychology, Virginia Tech, Blacksburg; 2 Fralin Biomedical Research Institute at VTC, Virginia Tech, Roanoke; 3 Department of Psychiatry, University of Pittsburgh School of Medicine, Pittsburgh, Pennsylvania; 4 School of Psychological and Cognitive Sciences, Beijing Key Laboratory of Behavior and Mental Health, PKU-IDG/McGovern Institute for Brain Research, Peking-Tsinghua Center for Life Sciences, Peking University, Beijing, China; 5 Virginia Tech-Wake Forest University School of Biomedical Engineering and Sciences, Blacksburg

## Abstract

**Importance:**

Major depressive disorder is prevalent and impairing. Parsing neurocomputational substrates of reinforcement learning in individuals with depression may facilitate a mechanistic understanding of the disorder and suggest new cognitive therapeutic targets.

**Objective:**

To determine associations among computational model–derived reinforcement learning parameters, depression symptoms, and symptom changes after treatment.

**Design, Setting, and Participants:**

In this mixed cross-sectional–cohort study, individuals performed reward and loss variants of a probabilistic learning task during functional magnetic resonance imaging at baseline and follow-up. A volunteer sample with and without a depression diagnosis was recruited from the community. Participants were assessed from July 2011 to February 2017, and data were analyzed from May 2017 to May 2021.

**Main Outcomes and Measures:**

Computational model–based analyses of participants’ choices assessed a priori hypotheses about associations between components of reward-based and loss-based learning with depression symptoms. Changes in both learning parameters and symptoms were then assessed in a subset of participants who received cognitive behavioral therapy (CBT).

**Results:**

Of 101 included adults, 69 (68.3%) were female, and the mean (SD) age was 34.4 (11.2) years. A total of 69 participants with a depression diagnosis and 32 participants without a depression diagnosis were included at baseline; 48 participants (28 with depression who received CBT and 20 without depression) were included at follow-up (mean [SD] of 115.1 [15.6] days). Computational model–based analyses of behavioral choices and neural data identified associations of learning with symptoms during reward learning and loss learning, respectively. During reward learning only, anhedonia (and not negative affect or arousal) was associated with model-derived learning parameters (learning rate: posterior mean regression β = −0.14; 95% credible interval [CrI], −0.12 to −0.03; outcome sensitivity: posterior mean regression β = 0.18; 95% CrI, 0.02 to 0.37) and neural learning signals (moderation of association between striatal prediction error and expected value signals: *t*_97_ = −2.10; *P* = .04). During loss learning only, negative affect (and not anhedonia or arousal) was associated with learning parameters (outcome shift: posterior mean regression β = −0.11; 95% CrI, −0.20 to −0.01) and disrupted neural encoding of learning signals (association with subgenual anterior cingulate prediction error signals: *r* = −0.28; *P* = .005). Symptom improvement following CBT was associated with normalization of learning parameters that were disrupted at baseline (reward learning rate: posterior mean regression β = 0.15; 90% CrI, 0.001 to 0.41; loss outcome shift: posterior mean regression β = 0.42; 90% CrI, 0.09 to 0.77).

**Conclusions and Relevance:**

In this study, the mapping of reinforcement learning components to symptoms of major depression revealed mechanistic features associated with these symptoms and points to possible learning-based therapeutic processes and targets.

## Key Points

**Question:**

Are depression symptoms associated with features of reinforcement learning, and if so, is treatment-related symptom change associated with learning changes?

**Findings:**

In this mixed cross-sectional–cohort study including 101 participants, participants with and without depression completed a probabilistic learning task during functional magnetic resonance imaging; participants with depression were reassessed after cognitive behavioral therapy (CBT). Computational model–based analyses of behavioral choices and neural data identified associations of learning with symptoms during reward learning and loss learning, respectively; symptom improvement following CBT was associated with normalization of learning parameters.

**Meaning:**

Mapping reinforcement learning processes to symptoms of depression reveals mechanistic features of these symptoms and points to possible learning-based therapeutic processes and targets.

## Teaser

This mixed cross-sectional–cohort study evaluates associations among computational model–derived reinforcement learning parameters, depression symptoms, and symptom changes after cognitive behavioral therapy.

## Introduction

Major depressive disorder affects approximately 7% of people in the US each year^1^ and is among the highest causes of disability in the world.^2^ However, characterizing and treating major depressive disorder is hampered by significant symptom heterogeneity.^3^ Recent paradigms^4^ suggest that moving beyond diagnostic status to focus on associations of major depression’s central impairments of anhedonia and negative affect^5^ with neurocomputational substrates of reinforcement learning^6,7^ may more precisely identify disrupted processes in individuals with depression and novel treatment targets. To that end, we sought to investigate the association of computational model–derived learning impairments with canonical depression symptoms and tested the translational relevance of these impairments by examining their responsiveness to symptom change after cognitive behavioral therapy (CBT).

According to computational formalizations of reinforcement learning, expectations about the outcomes of choices are updated based on prediction errors.^7,8,9^ This framework separates learning into computationally derived components associated with behaviorally and neurobiologically distinguishable processes (eg, outcome valuation vs expectation updating^10,11^). Computational model–based analyses differentiate and quantify these learning processes as model parameters that may then be associated with symptoms at the individual level. For depression, this approach has the potential to identify sources of disrupted responsivity to rewards and losses, including altered value updating following feedback, relative valuation of positive or negative feedback, and changes in overall valuation of outcomes.^6^

Such learning processes may be affected by both stimulus valence (eg, learning from rewards vs losses) and depression symptoms.^6,12,13,14,15,16,17,18,19,20,21^ With regard to the canonical symptoms of depression, anhedonia (ie, reduced experience of pleasure) affects reward learning more than depression as a whole,^10,22,23,24,25^ while negative affect, characterized by subjective distress and negative cognitions, may be associated with altered loss and error processing.^12,13,18,20^ This link between symptom clusters and neurobehavioral alterations is consistent with other work showing symptom, not diagnosis, effects.^26,27,28^ Initial findings combining these literatures^13,17,18,29^ suggest valence-dependent roles of learning anomalies in depression, but to our knowledge, no study has fully examined which reward learning and loss learning processes are associated with the core depressive symptoms of anhedonia and negative affect.

Demonstrating sensitivity to symptom change is critical to establishing the translational relevance of biobehavioral markers of psychiatric illness.^30,31^ Some evidence suggests that successful depression treatment may normalize reward responses and, in youth, reduce overresponsivity to punishments,^32,33,34^ but how these changes are associated with baseline impairments and whether they map onto mechanistic learning processes are unclear. To address these issues, we examined participants with depression who engaged in CBT, an efficacious psychotherapy theorized to reduce symptoms in part through changing learning,^35,36,37^ and tested whether symptom improvement following CBT was associated with changes in learning components. Given previous work indicating correlated decreases in all symptom measures after CBT,^38,39^ these analyses focused on learning parameter changes associated with overall symptom change rather than specific symptom subscales following CBT.

To summarize, we examined participants with and without a depression diagnosis performing reward and loss variants of a learning task while undergoing functional magnetic resonance imaging; a subset of the participants with depression was retested after completing CBT. We hypothesized that distinct processes in reward and loss learning, captured by computational model–derived parameters measuring aspects of updating and valuation and their corresponding neural signals, would be associated with symptoms of anhedonia and negative affect, respectively. Moreover, we posited that changes in these reward and loss learning parameters would be correlated with symptom improvement after treatment.

## Methods

### Study Design and Participants

A total of 101 participants were recruited via community advertisements from southwest Virginia and Houston, Texas. The Baylor College of Medicine and Virginia Tech institutional review boards approved study procedures, and all participants provided written informed consent after receiving a complete description of the study. A total of 69 participants with depression had a primary *DSM*-*IV*^40^ diagnosis of major depressive disorder or dysthymia, assessed with the Structured Clinical Interview for *DSM*-*IV*^41^; 32 nonpsychiatric control participants had no history of any *DSM* disorder. Participants completed a battery of measures, including the Mood and Anxiety Symptom Questionnaire (MASQ),^42^ a validated self-report measure of symptom clusters of anhedonia (anhedonic depression subscale), negative affect (general distress subscale), and arousal (anxious arousal subscale), which were the primary symptoms of interest, as well as the Beck Depression Inventory-II^43^ to assess overall depression severity, the Wechsler Test of Adult Reading^44^ to estimate verbal IQ, and a demographics questionnaire. The eMethods and eTable 8 in the Supplement contains further details about participants and measures. This study followed the Strengthening the Reporting of Observational Studies in Epidemiology (STROBE) reporting guideline.

### Reinforcement Learning Task

**Figure 1.**
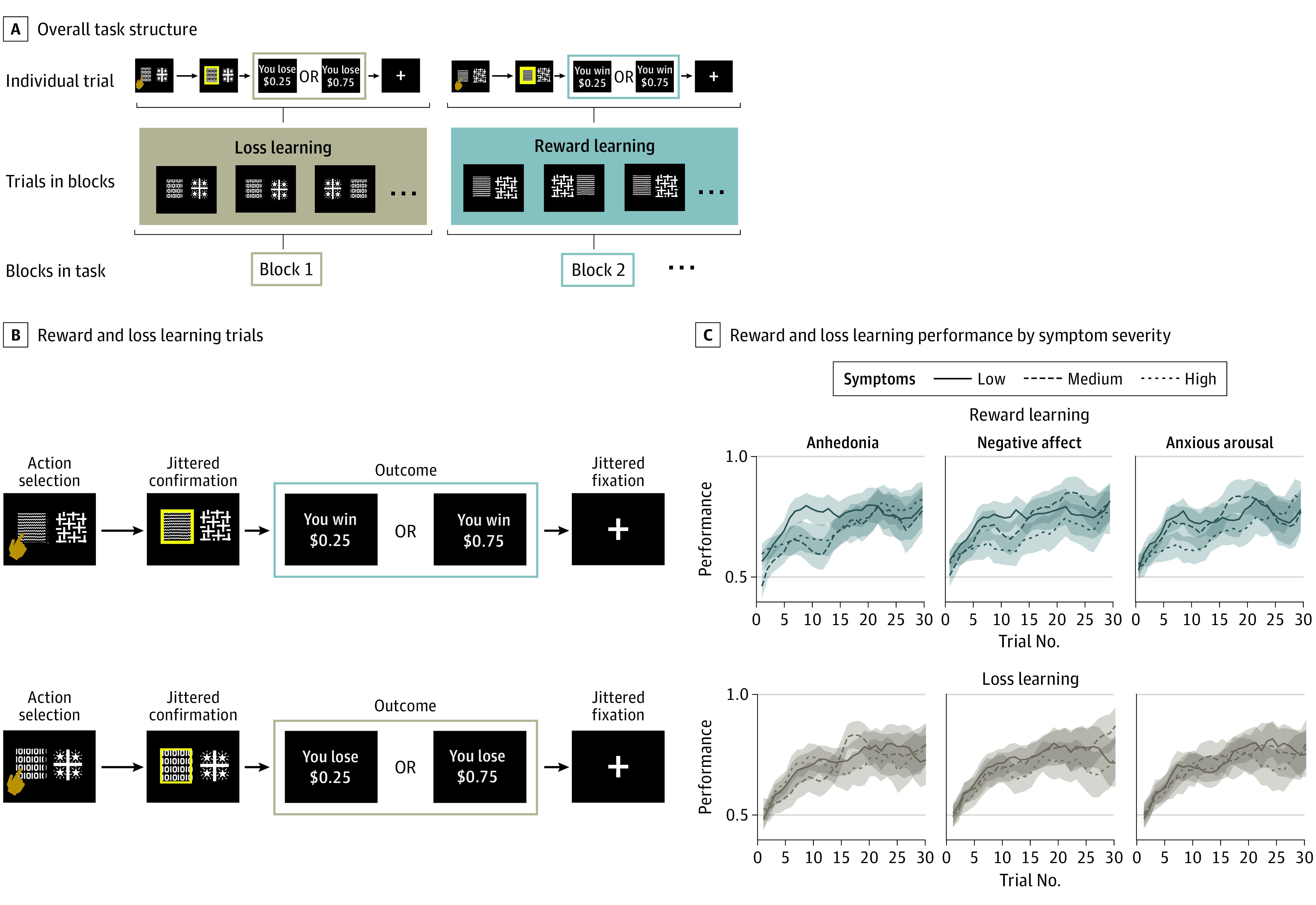
Reinforcement Learning Task Schematic and Overall Learning Curves A, Overall task structure. Participants completed trials of the same valence (reward learning or loss learning) with stimuli remaining consistent throughout a block. Once participants had learned stimulus contingencies for a block, a new block began with different stimuli and the other valence (loss or reward). The task ended when participants had at least 25 correct trials and 50 total trials for both reward learning and loss learning (median number of trials completed, 50). B, Schematic depiction of reward learning and loss learning trials. On each trial, participants were presented with 2 abstract stimuli. After choosing a stimulus, the chosen option was highlighted for a brief period and then an outcome (monetary reward or loss) was shown. Participants learned which option led to the better (75% probability of high reward or low loss) outcome. The task involved blocks consisting of trials with all reward (top) and all loss (bottom) outcomes. C, Reward learning and loss learning performance by symptom severity among all 101 participants. Performance was quantified as proportion of choices that were the better option. Over time, participants showed learning (running mean over 3 trials; averaged over all blocks by valence). Top panels comprise reward learning blocks while bottom panels comprise loss learning blocks. Behavior is separated by anhedonia, negative affect, and anxious arousal symptom severity, with participants with symptoms in the lowest tercile marked by a solid line, the middle tercile by a dashed line, and the highest tercile with a dotted line. The lines indicate mean scores, and the shaded areas indicate SEs.

Participants completed reward and loss variants of a probabilistic operant learning task (Figure 1A) with the goal of learning which of 2 options was more likely to lead to a higher outcome (larger reward in reward learning blocks or smaller loss in loss learning blocks; Figure 1B)^45^ while undergoing functional magnetic resonance imaging. The task was presented in pseudorandomized blocks of trials consisting of all reward outcomes or all loss outcomes (learning curves by baseline symptoms shown in Figure 1C). The eMethods in the Supplement contains further task design details.

### Behavioral Analyses at Baseline

Model-based analyses used reinforcement learning models to test hypotheses about potential sources of learning disruptions in participants with depression (updating, relative valuation of more negative or positive outcomes, and overall valuation changes^6,10,46^). The best-fitting model separated learning by valence (reward vs loss) and included 3 free parameters for both reward learning and loss learning: learning rate (α), which indexed the degree of updating based on prediction error; outcome sensitivity (ρ), which multiplicatively scaled more extreme outcome values, resulting in differential valuation of large vs small outcome values; and outcome shift (τ), which linearly shifted all outcome values, resulting in an overall positive or negative valuation bias. Model validation showed that parameters could be independently estimated, were associated with model-agnostic behavior, had good split-half and test-retest reliability, and were stable over time in participants without depression (eMethods and eFigures 2 to 4 and 12 in the Supplement).

### Baseline Imaging Analyses

Parametric regressors of interest in first-level imaging analyses used prediction error δ*_t_* at outcome and chosen expected value *Q_t_* at stimulus onset. Analyses focused on meta-analytically–defined^47^ regions of interest (ROIs) in ventral striatum and ventromedial prefrontal cortex/subgenual anterior cingulate cortex, primary brain regions implicated in reinforcement learning.^48^ Functional magnetic resonance imaging data collection, preprocessing, and further analysis information are contained in the eMethods in the Supplement.

### Behavioral Analyses of Changes in Symptoms and Learning Parameters Following Cognitive Behavioral Therapy

A total of 28 participants with depression who elected to engage in a 12-week course of standard, manual-guided CBT^49^ were assessed following CBT completion with the same procedures described above (eFigure 10 in the Supplement for a diagram of participant flow). These analyses assessed associations between symptom improvement, well-established with CBT,^35,50^ and learning parameters. Further details on CBT, CBT-related analyses, and symptom-independent parameter change (including stability of parameters in 20 nonpsychiatric control participants) can be found in eMethods and eFigures 8 and 9 in the Supplement.

### Statistical Analysis

Associations between symptoms and learning parameters were estimated within models fit to participants’ choices. Models were fit using hierarchical Bayesian estimation^51^ with data from all participants in the pertinent analysis; significant associations between symptoms and learning parameters were defined as a 95% posterior credible interval (CrI) of the regression coefficient for the association of symptom with learning parameter excluding 0, analogous to a frequentist α of .05. The posterior mean, the posterior mean divided by the posterior standard deviation (approximate standardized regression β), and 95% CrIs are reported. To control for false-positive rates, all associations were assessed at baseline (and similarly for treatment analyses) using hierarchical modeling^52^; additional Bayesian error control^53^ approaches restricted experimentwise error to 5%. Simulation-based power analyses, assuming 80% power, indicated we were powered to detect small effect sizes in regression analyses between symptom severity measures and learning parameters involving all 101 participants and medium effect sizes in analyses of 69 participants with depression only. See the eMethods in the Supplement for modeling details.

To test the association of neural activation with symptom measures, regressor-related blood oxygenation level–dependent activity (ROI values) was correlated with symptom measures using linear regressions and Pearson correlations. Following previous literature, for reward learning, the moderation of expected value and prediction error–related activity by symptoms was evaluated by testing the interaction of prediction error neural signal and symptoms on expected value neural signal in striatal ROIs.^25^ Frequentist analyses used an α level of .05 from 2-tailed tests.

Effects of symptom change following CBT (time × symptom analysis) were assessed as the association between changes in learning parameters and symptom changes (improvement pretreatment to posttreatment, similar to a mixed-effects analysis with 2 time points) in the participants with depression, controlling for initial symptom severity. Like baseline analyses, the CrI (set to 90% for significance to reflect directionality of hypotheses), the mean value of this association, and the standardized mean value as a measure of effect size are reported. As symptom subscale changes with CBT are typically highly intercorrelated,^38,39^ primary analyses focused on changes in learning parameters against changes in overall symptoms; significant associations were then examined with exploratory analyses within anhedonia, negative affect, and arousal subscales. Frequentist analyses involving symptom change used an α level of .05 and 1-tailed tests based on directional hypotheses. Analyses were carried out in R version 3.6.0 (The R Foundation) and Stan version 2.19 (using the rstan package in R).

## Results

### Participant Characteristics at Baseline

Of 101 included adults, 69 (68.3%) were female, and the mean (SD) age was 34.4 (11.2) years. A total of 69 participants with a depression diagnosis and 32 participants without a depression diagnosis were included at baseline; 48 participants (28 with depression who received CBT and 20 without depression) were included at follow-up (mean [SD] of 115.1 [15.6] days). Clinical and demographic data are reported in Table 1. As expected, participants with a depression diagnosis had elevated symptoms but did not differ from participants without depression on estimated IQ, age, or self-reported gender.

**Table 1.** Baseline Clinical and Demographic Data

Characteristic	Mean (SE)		Group comparison	*P* value
	No depression (n = 32)	Depression (n = 69)^a^		
Age	32.3 (1.9)	35.4 (1.4)	*t*_99_ = −1.36	.18
Female, No. (%)	20 (62.5)	49 (71.0)	χ^2^_1_ = 0.39	.53
Estimated IQ	107.3 (2.3)	107.6 (1.3)	*t*_99_ = −0.10	.92
Depression severity^b^	2.0 (0.5)	31.2 (1.0)	*t*_99_ = −20.2	<.001
Anhedonia^c^	44.8 (1.7)	83.8 (1.2)	*t*_99_ = −19.1	<.001
Negative affect^d^	23.1 (1.1)	46.0 (1.1)	*t*_99_ = −13.2	<.001
Anxious arousal^e^	18.6 (0.4)	26.5 (0.9)	*t*_99_ = −5.96	<.001

^a^ A total of 63 participants had a diagnosis of major depressive disorder, 3 had a diagnosis of dysthymia, and 3 had both diagnoses.

^b^ Beck Depression Inventory-II total.

^c^ Mood and Anxiety Symptom Questionnaire anhedonic depression subscale.

^d^ Mood and Anxiety Symptom Questionnaire general distress subscale.

^e^ Mood and Anxiety Symptom Questionnaire anxious arousal subscale.

### Baseline Model-Based Analyses

We tested associations of computational model–derived learning parameters (learning rate, outcome sensitivity, and outcome shift) with symptom severity (MASQ subscales of anhedonia, negative affect, and anxious arousal, tested in separate regressions) during reward and loss learning (Table 2). Follow-up analyses simultaneously assessed all 3 MASQ subscales in the same analysis and tested depression diagnosis and Beck Depression Inventory-II score as a measure of overall depression severity (eResults in the Supplement). Analyses were carried out across all participants and within participants with a diagnosis of depression only.

**Table 2.** Associations Between Symptoms and Reinforcement Learning Parameters at Baseline and Changes With Symptom Change After Treatment

Population	Context	Symptom cluster	Parameter	Regression β	
				Mean (CrI)	Standardized mean
Participants with depression, baseline	Reward	Anhedonia	Learning rate	−0.14 (95% CrI, −0.12 to −0.03)^a^	−2.59^a^
			Outcome sensitivity	0.18 (95% CrI, 0.02 to 0.37)^a^	2.01^a^
			Outcome shift	−0.03 (95% CrI, −0.13 to 0.06)	−0.62
		Negative affect	Learning rate	−0.08 (95% CrI, −0.11 to 0.08)	−1.10
			Outcome sensitivity	−0.02 (95% CrI, −0.18 to 0.13)	−0.28
			Outcome shift	−0.02 (95% CrI, −0.11 to 0.07)	−0.46
		Arousal	Learning rate	0.01 (95% CrI, −0.09 to 0.18)	0.06
			Outcome sensitivity	−0.05 (95% CrI, −0.12 to 0.11)	−0.69
			Outcome shift	0.01 (95% CrI, −0.08 to 0.09)	0.25
All participants, baseline	Loss	Anhedonia	Learning rate	0.03 (95% CrI, −0.05 to 0.15)	0.61
			Outcome sensitivity	0.16 (95% CrI, −0.21 to 0.55)	0.80
			Outcome shift	−0.05 (95% CrI, −0.14 to 0.05)	−0.96
		Negative affect	Learning rate	0.05 (95% CrI, −0.04 to 0.16)	0.09
			Outcome sensitivity	0.33 (95% CrI, −0.04 to 0.71)	1.76
			Outcome shift	−0.11 (95% CrI, −0.20 to −0.01)^a^	−2.17^a^
		Arousal	Learning rate	0.02 (95% CrI, −0.05 to 0.14)	0.41
			Outcome sensitivity	0.27 (95% CrI, −0.08 to 0.59)	1.56
			Outcome shift	−0.08 (95% CrI, −0.17 to 0.01)	−1.68
Participants with depression, pre- vs posttreatment	Reward	Total (change)	Learning rate (change)	0.15 (90% CrI, 0.001 to 0.41)^a^	1.71^a^
			Outcome sensitivity (change)	−0.30 (90% CrI, −0.94 to 0.38)	−0.74
			Outcome shift (change)	−0.06 (90% CrI, −0.39 to 0.25)	−0.29
	Loss	Total (change)	Learning rate (change)	−0.03 (90% CrI, −0.02 to 0.16)	−0.46
			Outcome sensitivity (change)	−0.38 (90% CrI, −1.52 to 0.79)	−0.53
			Outcome shift (change)	0.42 (90% CrI, 0.09 to 0.77)^a^	2.03^a^

Abbreviation: CrI, credible interval.

^a^ *P* < .05.

#### Reward Learning

##### Behavioral

**Figure 2.**
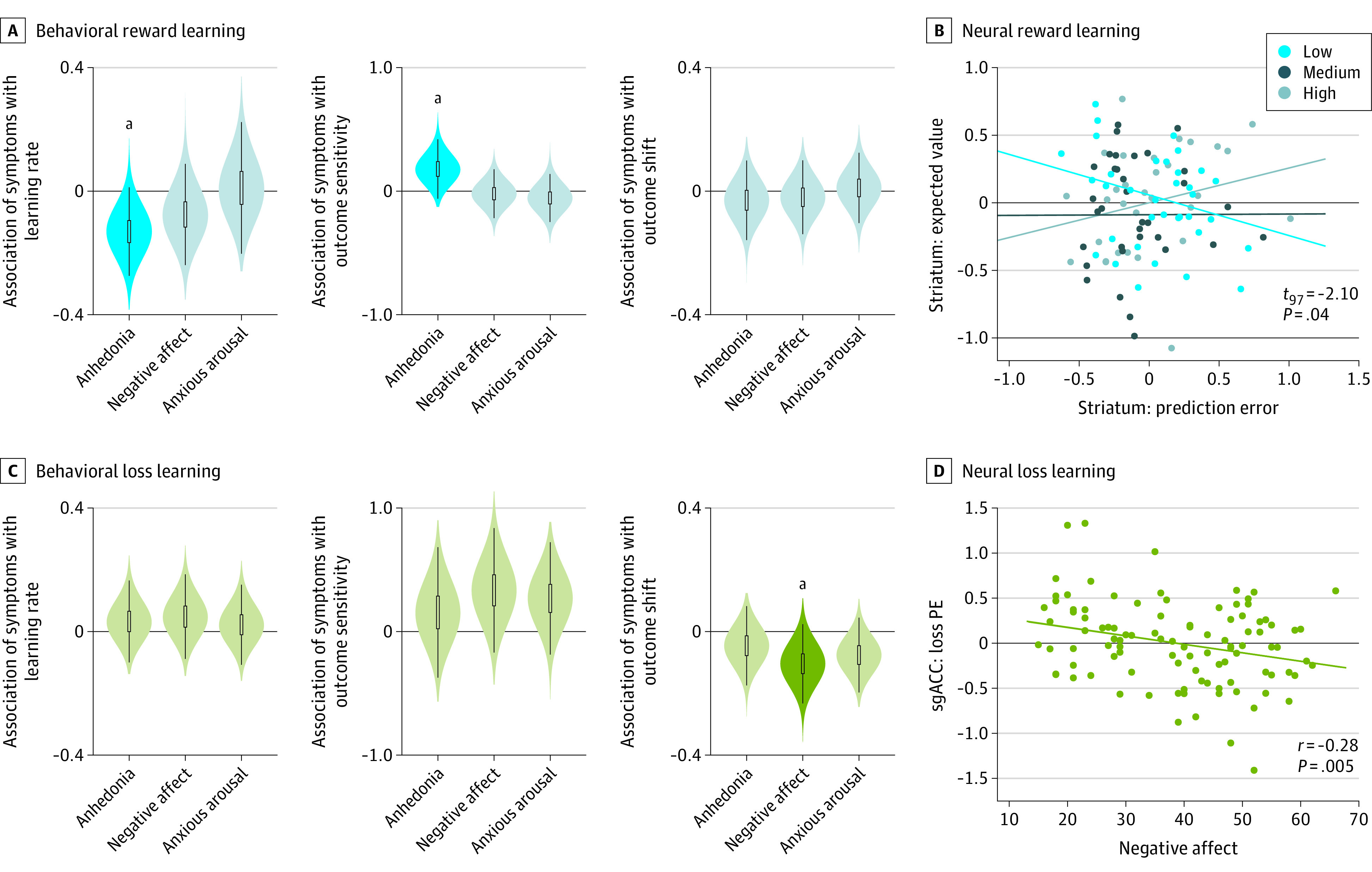
Association of Behavioral and Neural Indicators of Reinforcement Learning With Symptoms of Depression A, Behaviorally, in reward learning in participants with depression, greater anhedonia is associated with lower learning rate and higher outcome sensitivity. Violin plots indicate posterior distribution of the association between the behavioral learning parameter and symptom measure. Anhedonia refers to the Mood and Anxiety Symptom Questionnaire anhedonia scale, negative affect to the Mood and Anxiety Symptom Questionnaire general distress scale, and anxious arousal to the Mood and Anxiety Symptom Questionnaire anxious arousal scale. Posterior distributions are from regressions testing each scale separately for associations with parameters; results were similar when testing all scales in the same analysis. B, Neurally, across all participants, anhedonia moderated the association between striatal prediction error (PE) responses and expected value. Low, medium, and high anhedonia were based on tercile split for the right striatum region of interest activation to PE (x-axis) and expected value (y-axis). Lines indicate regression lines for each group to illustrate moderation of the PE to expected value association by anhedonia. C, Behaviorally, in loss learning across all participants, greater negative affect was associated with more negative outcome shift. Violin plots indicate posterior distribution of the association between behavioral learning parameter and symptom measure. Posterior distributions are from regressions testing each scale separately for associations with parameters; results were similar when testing all scales in the same analysis. D, Neurally, negative affect was negatively associated with subgenual anterior cingulate cortex (sgACC) signaling of PE at the time of outcome receipt. Dots indicate individual participants’ negative affect severity vs sgACC region of interest activation to PE; the regression line indicates the overall negative association. ^a^*P* < .05.

During reward learning, in participants with depression, greater anhedonia was associated with reduced learning rate, indicating slower updating of reward values with increased anhedonia and greater outcome sensitivity parameter values (learning rate: posterior mean regression β = −0.14; 95% CrI, −0.12 to −0.03; outcome sensitivity: posterior mean regression β = 0.18; 95% CrI, 0.02 to 0.37; Figure 2A). These associations were apparent with anhedonia and absent with negative affect, arousal, or depression diagnosis. No measures were associated with changes in the outcome shift parameter. Results were similar when assessing all MASQ scales in the same analysis (eResults in the Supplement). When including participants without depression, these associations were not present (association of anhedonia and learning rate: mean, −0.05; 95% CrI, −0.01 to 0.07; standardized mean, 0.95; outcome sensitivity: mean, −0.03; 95% CrI, −0.15 to 0.09; standardized mean, −0.50). Model-agnostic results (eResults and eFigures 1 and 5 in the Supplement) were also consistent with an association between reward learning and anhedonia in participants with clinical levels of depression.

##### Neural

Reward prediction error and expected value signals did not vary with any symptom measure or depression diagnosis (meta-analytically defined striatal or ventromedial prefrontal cortex ROIs or exploratory whole-brain analysis; eFigure 6 and eTables 1 to 4 in the Supplement). Following previous work, to assess if anhedonia disrupted associations among otherwise intact striatal signals,^25,54^ we investigated associations between prediction error (at outcome) and expected value (at choice) in ventral striatum. In line with this previous work, anhedonia moderated the association between expected value and prediction error signals (interaction: *t*_97_ = −2.10; *P* = .04; Figure 2B), which was not associated with learning rate differences.

#### Loss Learning

##### Behavioral

During loss learning, a different pattern of associations emerged such that negative affect severity was associated with more negative outcome shift parameter values, indicating more negative valuation of losses (outcome shift: posterior mean regression β = −0.11; 95% CrI, −0.20 to −0.01; Figure 2C). This association was present in all participants, regardless of depression diagnosis, and was not observed with anhedonia, arousal, or depression diagnosis. Associations were similar when assessing all MASQ scales simultaneously. No symptom subscales were associated with loss learning rate or outcome sensitivity parameters.

##### Neural

Prediction error activity in the subgenual anterior cingulate cortex ROI was negatively associated with negative affect (*r* = −0.28; *P* = .005; Figure 2D), with no differences in striatal activity or expected value signals. Exploratory follow-up analyses (eMethods, eFigure 7, and eTables 5 to 7 in the Supplement) suggested reduced subgenual anterior cingulate cortex representation of outcome value in participants with high negative affect drove this association. Expected value and the association between expected value and prediction error were unrelated to symptom measures during loss learning.

### Associations Between Learning Parameter Changes and Symptom Changes Following Cognitive Behavioral Therapy

The association of baseline symptoms with learning parameters suggested the translational potential of reinforcement learning processes beyond descriptive characterizations of depression. We thus sought to assess whether these altered learning processes were associated with symptom changes following CBT in participants with depression.

**Figure 3.**
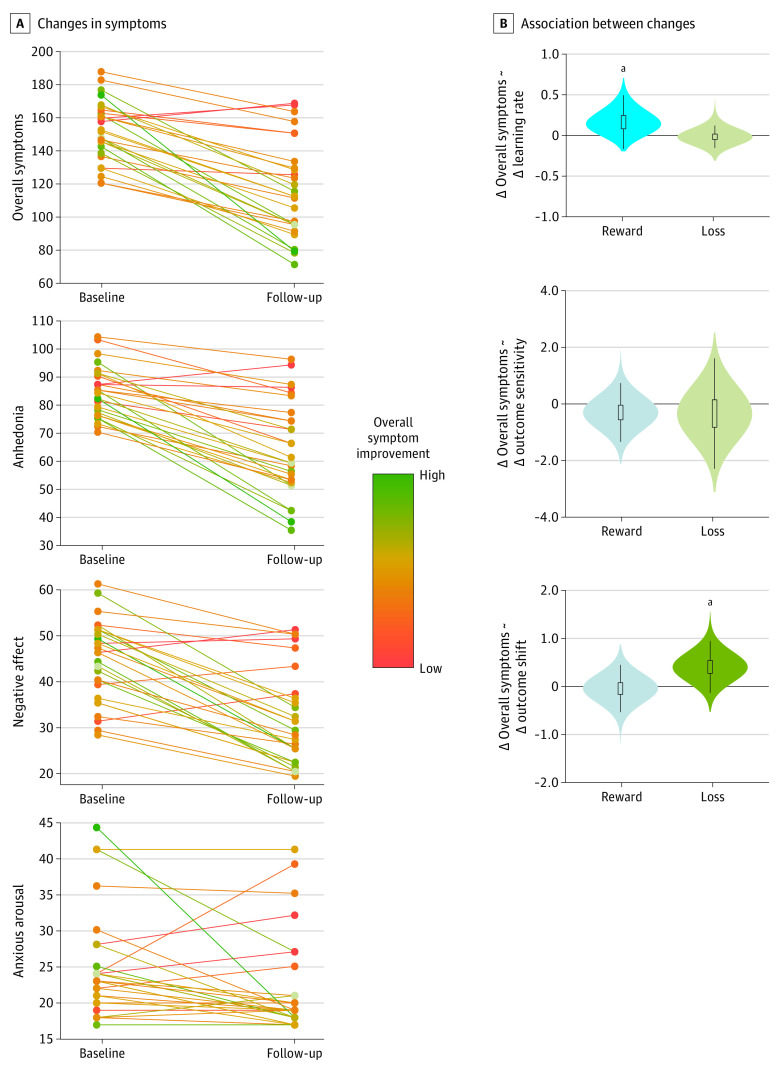
Changes in Depression Symptoms and Behavioral Reinforcement Learning Parameters With Cognitive Behavioral Therapy A, Changes in symptoms from pretreatment to posttreatment for 28 patients. Total symptoms (sum of Mood and Anxiety Symptom Questionnaire anhedonia, general distress, and anxious arousal scales), anhedonia (Mood and Anxiety Symptom Questionnaire anhedonia scale), negative affect (Mood and Anxiety Symptom Questionnaire general distress scale), and anxious arousal (Mood and Anxiety Symptom Questionnaire anxious arousal scale) all decreased on average from pretreatment to posttreatment with cognitive behavioral therapy, but with large heterogeneity in treatment response across participants. Individual lines of symptom change are colored by the degree of overall symptom improvement (green: high improvement; orange: low improvement) to illustrate consistent rates of improvement across individual subscales. B, Association between changes in reinforcement learning parameters and symptom changes with cognitive behavioral therapy. Increases in learning rate were correlated with symptom improvement for reward learning, and increases in outcome shift were correlated with symptom improvement for loss learning. Violin plots indicate posterior distribution of the association between changes in behavioral learning parameter and percentage change in symptoms. ^a^*P* < .05.

As expected, after CBT, participants showed large mean decreases in all symptoms (Figure 3A). Consistent with the literature,^55^ participants showed heterogenous degrees of change, enabling investigation of individual differences in symptom change (Figure 3A). As within-participant changes in symptom measures were highly correlated (eg, correlation between change in anhedonia and negative affect: *r* = 0.62; *P* < .001), analyses focused on overall improvement (summing anhedonia + negative affect + arousal scales; if significant, exploratory analyses, reported in eTable 9 in the Supplement, focused on individual subscale change) as associated with changes in learning parameters (outcome sensitivity, outcome shift, and learning rate during reward learning and loss learning).

#### Behavioral

As described above, at baseline, reward learning rate was negatively correlated and reward outcome sensitivity positively correlated with anhedonia. Increases in reward learning rate following CBT were significantly associated with overall symptom improvement, including improved anhedonia (reward learning rate: posterior mean regression β = 0.15; 90% CrI, 0.001 to 0.41; Figure 3B; Table 2). Changes in reward outcome sensitivity were not significantly associated with overall symptom improvement. Changes in reward outcome shift, unrelated to symptoms at baseline, were also not associated with symptom change.

During loss learning at baseline, the outcome shift learning parameter showed a negative association with negative affect. Increases in loss outcome shift after CBT were significantly associated with overall symptom improvement, including improved negative affect (loss outcome shift: posterior mean regression β = 0.42; 90% CrI, 0.09 to 0.77; Figure 3B; Table 2). Changes in loss learning rate and outcome sensitivity, which were unrelated to symptoms at baseline, were also not associated with changes in overall symptoms.

#### Neural

For reward learning, at baseline, anhedonia moderated associations between prediction error and expected value signaling in striatum; following CBT, participants with depression with high anhedonia showed a significant change in the correlation between ventral striatum signaling to prediction error and expected value from pretreatment to posttreatment (Fisher *r* to *z* = 1.65; 1-tailed *P* = .05; eFigure 11A in the Supplement). In participants without depression, this correlation was stable across time (*z* = 0.46; *P* = .65), and the overall interaction across all participants was significant (interaction of baseline anhedonia with the association between changes in expected value and prediction error signals in ventral striatum: *t*_43_ = 1.85; 1-tailed *P* = .04), indicating a shift only in participants with high anhedonia. For loss learning, changes in prediction error signaling in subgenual anterior cingulate cortex (related to negative affect at baseline) were not associated with improvements in negative affect (eFigure 11B in the Supplement).

## Discussion

Here, we used a computational model of reinforcement learning to distinguish among learning processes in participants with and without depression and showed, across neural and behavioral levels, associations of anhedonia with reduced updating but greater differentiation of rewards (captured by reward learning rate and outcome sensitivity parameters, respectively) and of negative affect with more negative valuation of losses (captured by the loss outcome shift parameter). Broad symptom improvement after CBT, including improved anhedonia and negative affect, was associated with normalization of reward learning rate and loss outcome shift disruptions, respectively.

Similar to other studies with large patient samples,^25,54,56^ for reward learning, we found no support of a reduction in valuation with anhedonia but rather a moderation of neural expected value prediction error correlations in ventral striatum. These results suggest that highly anhedonic individuals paradoxically process large rewards as more rewarding but then fail to update future reward expectations and are consistent with previous findings of increased immediate responsivity but reduced long-term effects of rewards in individuals with depression.^57,58^ During loss learning, participants higher in negative affect showed more negative valuation of outcomes and no learning rate variation, suggesting that maladaptive overresponsivity to losses observed in individuals with depression^12,15,20^ may be due to valuing negative feedback more negatively and not to overadjusting following negative feedback. Overall, our findings of altered learning processes at baseline show that depression impairs aspects of reward learning and loss learning but in potentially distinct ways: poor reward learning is because of slow updating while disrupted loss learning results from pessimistic valuation of outcomes. Of interest, the increased reward outcome sensitivity in participants with depression with greater anhedonia is discrepant from previous reports^10,59^ that included participants lower in anhedonia or smaller patient samples not permitting dimensional analyses. Future studies are warranted to fully delineate the nature and impact of associations between anhedonia and outcome sensitivity in depression.

Of clinical importance is whether reinforcement learning processes are sensitive to treatment, which would indicate a potential causal relationship between learning changes and symptom improvement. We indeed found that symptom change following CBT was correlated with remediation of altered learning parameters and neural responses. Specifically, greater symptom improvement was accompanied by increased reward learning rate, a normalized association between neural signals of expected value and prediction error during reward learning, and a more positive outcome shift during loss learning. The learning changes have conceptual overlap with CBT’s focus on challenging negative evaluations and reflecting on outcomes of pleasurable activities.^49^ These associations suggest that model-derived learning parameters go beyond describing alterations at baseline and are sensitive to treatment-induced changes in symptoms. The data may thus inform the future development and testing of symptom-based or parameter-based therapies that directly target behavioral or neural circuits involved in reinforcement learning. Model-derived learning parameters may also be used to improve outcomes and tailor extant treatments to individual symptom presentations (eg, focusing on updating reward expectations in patients high in anhedonia vs more positive valuation of negative outcomes in patients high in negative affect).

### Limitations

The limitations of this work warrant attention. First, although our sample size was comparatively large, even larger samples would ensure adequate power to detect smaller effects, particularly with more conservative Bayesian multilevel analyses,^53,60^ and to statistically dissociate changes in specific symptom clusters after treatment. Second, while comparable with other work of this scope, our exclusion rate owing to issues with scanning or behavioral data was relatively high and may affect the generalizability of results. Indeed, although participants with depression were not excluded more often than those without depression, excluded participants did have lower estimated IQ. In addition, larger, more clinically heterogenous samples may be needed to detect symptom subscale–specific changes or reward outcome sensitivity changes following CBT. Future work may also clarify the specificity and sensitivity of learning parameters to change by comparing changes in associations of learning with symptoms between CBT and other treatments or natural variation in symptoms over time.

## Conclusions

In individuals with depression, associations between symptoms and disrupted valuation have long been hypothesized but difficult to dissociate. By parsing components of value-based learning in a large, well-characterized sample, we show that associations of learning with symptoms in individuals with depression are present and may vary by valence and learning process. The remediation of computational model–identified learning processes associated with symptom changes after CBT suggest a mechanistic role of learning disruptions in those with depression. More broadly, this work may provide a bridge between behaviorally oriented clinicians and computational (neuro)scientists toward novel integrative ways for understanding and treating depression.

## Notes

Supplement.
eMethods.

eResults.

eReferences.
**eFigure 1.** Association of overall performance with MDD diagnosis and symptom severity. **eFigure 2.** Model fit and parameter recovery. **eFigure 3.** Posterior distributions and individual means of parameters. **eFigure 4.** Association of model parameters with model-agnostic summaries of behavior. **eFigure 5.** Association between anhedonia and reward learning parameters by diagnosis. **eFigure 6.** Behavioral performance and neural reward signals by depression status and overall depression severity. **eFigure 7.** Differences in processing of loss outcomes by negative affect. **eFigure 8.** Stability of parameter estimates over time for control participants. **eFigure 9.** Parameter changes with time for participants with depression. **eFigure10.** Diagram of flow of participants through study, including optional CBT portion. **eFigure 11.** Neural responses associated with symptom improvement. **eFigure 12.** Schematic depiction of effects of outcome sensitivity and outcome shift on valuation. **eTable 1.** Reward prediction error, MDD group (n = 69). **eTable 2.** Reward expected value, MDD group (n = 69). **eTable 3.** Reward prediction error, controls without depression (n = 32). **eTable 4.** Reward expected value, controls without depression (n = 32). **eTable 5.** Loss outcome value correlated with negative affect (MASQ Mixed Distress subscale; n = 101). **eTable 6.** Loss outcome value, low negative affect participants (n = 52). **eTable 7.** Loss outcome value, high negative affect participants (n = 49). **eTable 8.** Depression diagnosis, specifier, severity, medications, and comorbid diagnoses for participants. **eTable 9.** Exploratory follow-up analyses of associations between symptom change and reinforcement learning parameters in participants with depression who completed CBT.

## References

[1] Kessler RC, Chiu WT, Demler O, Merikangas KR, Walters EE. Prevalence, severity, and comorbidity of 12-month *DSM*-*IV* disorders in the National Comorbidity Survey Replication. Arch Gen Psychiatry. 2005;62(6):617-627. doi:10.1001/archpsyc.62.6.617 15939839 PMC2847357

[2] World Health Organization. Depression and other common mental disorders: global health estimates. Accessed June 17, 2021. https://apps.who.int/iris/bitstream/handle/10665/254610/WHO-MSD-MER-2017.2-eng.pdf

[3] Stephan KE, Bach DR, Fletcher PC, . Charting the landscape of priority problems in psychiatry, part 1: classification and diagnosis. Lancet Psychiatry. 2016;3(1):77-83. doi:10.1016/S2215-0366(15)00361-2 26573970

[4] Insel T, Cuthbert B, Garvey M, . Research Domain Criteria (RDoC): toward a new classification framework for research on mental disorders. Am J Psychiatry. 2010;167(7):748-751. doi:10.1176/appi.ajp.2010.09091379 20595427

[5] Clark LA, Watson D. Tripartite model of anxiety and depression: psychometric evidence and taxonomic implications. J Abnorm Psychol. 1991;100(3):316-336. doi:10.1037/0021-843X.100.3.316 1918611

[6] Eshel N, Roiser JP. Reward and punishment processing in depression. Biol Psychiatry. 2010;68(2):118-124. doi:10.1016/j.biopsych.2010.01.027 20303067

[7] Schultz W, Dayan P, Montague PR. A neural substrate of prediction and reward. Science. 1997;275(5306):1593-1599. doi:10.1126/science.275.5306.1593 9054347

[8] Montague PR, Dayan P, Sejnowski TJ. A framework for mesencephalic dopamine systems based on predictive Hebbian learning. J Neurosci. 1996;16(5):1936-1947. doi:10.1523/JNEUROSCI.16-05-01936.1996 8774460 PMC6578666

[9] Sutton RS, Barto AG. Reinforcement Learning: An Introduction. MIT Press; 1998.

[10] Huys QJ, Pizzagalli DA, Bogdan R, Dayan P. Mapping anhedonia onto reinforcement learning: a behavioural meta-analysis. Biol Mood Anxiety Disord. 2013;3(1):12. doi:10.1186/2045-5380-3-12 23782813 PMC3701611

[11] Robinson OJ, Chase HW. Learning and choice in mood disorders: searching for the computational parameters of anhedonia. Comput Psychiatr. 2017;1(1):208-233. doi:10.1162/CPSY_a_00009 29400358 PMC5796642

[12] Chiu PH, Deldin PJ. Neural evidence for enhanced error detection in major depressive disorder. Am J Psychiatry. 2007;164(4):608-616. doi:10.1176/ajp.2007.164.4.608 17403974

[13] Cavanagh JF, Bismark AW, Frank MJ, Allen JJB. Multiple dissociations between comorbid depression and anxiety on reward and punishment processing: evidence from computationally informed EEG. Comput Psychiatr. 2019;3:1-17. doi:10.1162/CPSY_a_00024 31149639 PMC6515849

[14] Pizzagalli DA, Iosifescu D, Hallett LA, Ratner KG, Fava M. Reduced hedonic capacity in major depressive disorder: evidence from a probabilistic reward task. J Psychiatr Res. 2008;43(1):76-87. doi:10.1016/j.jpsychires.2008.03.001 18433774 PMC2637997

[15] Elliott R, Sahakian BJ, Herrod JJ, Robbins TW, Paykel ES. Abnormal response to negative feedback in unipolar depression: evidence for a diagnosis specific impairment. J Neurol Neurosurg Psychiatry. 1997;63(1):74-82. doi:10.1136/jnnp.63.1.74 9221971 PMC2169625

[16] Robinson OJ, Cools R, Carlisi CO, Sahakian BJ, Drevets WC. Ventral striatum response during reward and punishment reversal learning in unmedicated major depressive disorder. Am J Psychiatry. 2012;169(2):152-159. doi:10.1176/appi.ajp.2011.11010137 22420038 PMC5648982

[17] Rothkirch M, Tonn J, Köhler S, Sterzer P. Neural mechanisms of reinforcement learning in unmedicated patients with major depressive disorder. Brain. 2017;140(4):1147-1157. doi:10.1093/brain/awx025 28334960

[18] Luking KR, Pagliaccio D, Luby JL, Barch DM. Child gain approach and loss avoidance behavior: relationships with depression risk, negative mood, and anhedonia. J Am Acad Child Adolesc Psychiatry. 2015;54(8):643-651. doi:10.1016/j.jaac.2015.05.010 26210333 PMC4810675

[19] Kumar P, Goer F, Murray L, . Impaired reward prediction error encoding and striatal-midbrain connectivity in depression. Neuropsychopharmacology. 2018;43(7):1581-1588. doi:10.1038/s41386-018-0032-x 29540863 PMC5983542

[20] Aylward J, Valton V, Ahn W-Y, . Altered learning under uncertainty in unmedicated mood and anxiety disorders. Nat Hum Behav. 2019;3(10):1116-1123. doi:10.1038/s41562-019-0628-0 31209369 PMC6790140

[21] Dombrovski AY, Szanto K, Clark L, . Corticostriatothalamic reward prediction error signals and executive control in late-life depression. Psychol Med. 2015;45(7):1413-1424. doi:10.1017/S0033291714002517 25319564 PMC4380546

[22] Gradin VB, Kumar P, Waiter G, . Expected value and prediction error abnormalities in depression and schizophrenia. Brain. 2011;134(pt 6):1751-1764. doi:10.1093/brain/awr059 21482548

[23] Rothkirch M, Schmack K, Deserno L, Darmohray D, Sterzer P. Attentional modulation of reward processing in the human brain. Hum Brain Mapp. 2014;35(7):3036-3051. doi:10.1002/hbm.22383 24307490 PMC6869517

[24] Chase HW, Frank MJ, Michael A, Bullmore ET, Sahakian BJ, Robbins TW. Approach and avoidance learning in patients with major depression and healthy controls: relation to anhedonia. Psychol Med. 2010;40(3):433-440. doi:10.1017/S0033291709990468 19607754

[25] Greenberg T, Chase HW, Almeida JR, . Moderation of the relationship between reward expectancy and prediction error-related ventral striatal reactivity by anhedonia in unmedicated major depressive disorder: findings from the EMBARC study. Am J Psychiatry. 2015;172(9):881-891. doi:10.1176/appi.ajp.2015.14050594 26183698 PMC4858169

[26] Drysdale AT, Grosenick L, Downar J, . Resting-state connectivity biomarkers define neurophysiological subtypes of depression. Nat Med. 2017;23(1):28-38. doi:10.1038/nm.4246 27918562 PMC5624035

[27] Gillan CM, Kalanthroff E, Evans M, . Comparison of the association between goal-directed planning and self-reported compulsivity vs obsessive-compulsive disorder diagnosis. JAMA Psychiatry. 2020;77(1):77-85. doi:10.1001/jamapsychiatry.2019.2998 31596434 PMC6802255

[28] Young CB, Chen T, Nusslock R, Keller J, Schatzberg AF, Menon V. Anhedonia and general distress show dissociable ventromedial prefrontal cortex connectivity in major depressive disorder. Transl Psychiatry. 2016;6(5):e810. doi:10.1038/tp.2016.80 27187232 PMC5070048

[29] Harlé KM, Guo D, Zhang S, Paulus MP, Yu AJ. Anhedonia and anxiety underlying depressive symptomatology have distinct effects on reward-based decision-making. PLoS One. 2017;12(10):e0186473. doi:10.1371/journal.pone.0186473 29059254 PMC5653291

[30] Paulus MP, Huys QJM, Maia TV. A roadmap for the development of applied computational psychiatry. Biol Psychiatry Cogn Neurosci Neuroimaging. 2016;1(5):386-392. doi:10.1016/j.bpsc.2016.05.001 28018986 PMC5176268

[31] Mayberg HS. Modulating dysfunctional limbic-cortical circuits in depression: towards development of brain-based algorithms for diagnosis and optimised treatment. Br Med Bull. 2003;65:193-207. doi:10.1093/bmb/65.1.193 12697626

[32] Dichter GS, Felder JN, Petty C, Bizzell J, Ernst M, Smoski MJ. The effects of psychotherapy on neural responses to rewards in major depression. Biol Psychiatry. 2009;66(9):886-897. doi:10.1016/j.biopsych.2009.06.021 19726030 PMC3657763

[33] Heller AS, Johnstone T, Light SN, . Relationships between changes in sustained fronto-striatal connectivity and positive affect in major depression resulting from antidepressant treatment. Am J Psychiatry. 2013;170(2):197-206. doi:10.1176/appi.ajp.2012.12010014 23223803 PMC3563751

[34] Webb CA, Auerbach RP, Bondy E, Stanton CH, Appleman L, Pizzagalli DA. Reward-related neural predictors and mechanisms of symptom change in cognitive behavioral therapy for depressed adolescent girls. Biol Psychiatry Cogn Neurosci Neuroimaging. 2021;6(1):39-49. doi:10.1016/j.bpsc.2020.07.010 32948509 PMC7796984

[35] Beck AT. The current state of cognitive therapy: a 40-year retrospective. Arch Gen Psychiatry. 2005;62(9):953-959. doi:10.1001/archpsyc.62.9.953 16143727

[36] Roiser JP, Elliott R, Sahakian BJ. Cognitive mechanisms of treatment in depression. Neuropsychopharmacology. 2012;37(1):117-136. doi:10.1038/npp.2011.183 21976044 PMC3238070

[37] Dimidjian S, Barrera M Jr, Martell C, Muñoz RF, Lewinsohn PM. The origins and current status of behavioral activation treatments for depression. Annu Rev Clin Psychol. 2011;7:1-38. doi:10.1146/annurev-clinpsy-032210-104535 21275642

[38] Boumparis N, Karyotaki E, Kleiboer A, Hofmann SG, Cuijpers P. The effect of psychotherapeutic interventions on positive and negative affect in depression: a systematic review and meta-analysis. J Affect Disord. 2016;202:153-162. doi:10.1016/j.jad.2016.05.019 27262637

[39] Kring AM, Persons JB, Thomas C. Changes in affect during treatment for depression and anxiety. Behav Res Ther. 2007;45(8):1753-1764. doi:10.1016/j.brat.2007.02.001 17374361

[40] American Psychiatric Association. Diagnostic and Statistical Manual of Mental Disorders. 4th ed, text revision. American Psychiatric Association; 2000.

[41] First MB, Spitzer R, Gibbon M, Wiliams J. User’s Guide for the Structured Interview for DSM-IV Axis I Disorders—Research Version (SCID-I). Biometrics Research; 1996.

[42] Watson D, Weber K, Assenheimer JS, Clark LA, Strauss ME, McCormick RA. Testing a tripartite model: I. evaluating the convergent and discriminant validity of anxiety and depression symptom scales. J Abnorm Psychol. 1995;104(1):3-14. doi:10.1037/0021-843X.104.1.3 7897050

[43] Steer RA, Ball R, Ranieri WF, Beck AT. Dimensions of the Beck Depression Inventory-II in clinically depressed outpatients. J Clin Psychol. 1999;55(1):117-128. doi:10.1002/(SICI)1097-4679(199901)55:1<117::AID-JCLP12>3.0.CO;2-A 10100838

[44] Wechsler D. Wechsler Test of Adult Reading: WTAR. Psychological Corporation; 2001.

[45] Brown VM, Zhu L, Wang JM, Frueh BC, King-Casas B, Chiu PH. Associability-modulated loss learning is increased in posttraumatic stress disorder. Elife. 2018;7:e30150. doi:10.7554/eLife.30150 29313489 PMC5760201

[46] Chen C, Takahashi T, Nakagawa S, Inoue T, Kusumi I. Reinforcement learning in depression: a review of computational research. Neurosci Biobehav Rev. 2015;55:247-267. doi:10.1016/j.neubiorev.2015.05.005 25979140

[47] Chase HW, Kumar P, Eickhoff SB, Dombrovski AY. Reinforcement learning models and their neural correlates: an activation likelihood estimation meta-analysis. Cogn Affect Behav Neurosci. 2015;15(2):435-459. doi:10.3758/s13415-015-0338-7 25665667 PMC4437864

[48] Rangel A, Camerer C, Montague PR. A framework for studying the neurobiology of value-based decision making. Nat Rev Neurosci. 2008;9(7):545-556. doi:10.1038/nrn2357 18545266 PMC4332708

[49] Munoz RF, Miranda J. Individual Therapy Manual for Cognitive-Behavioral Treatment for Depression. RAND; 1996.

[50] DeRubeis RJ, Siegle GJ, Hollon SD. Cognitive therapy versus medication for depression: treatment outcomes and neural mechanisms. Nat Rev Neurosci. 2008;9(10):788-796. doi:10.1038/nrn2345 18784657 PMC2748674

[51] Ahn W-Y, Haines N, Zhang L. Revealing neurocomputational mechanisms of reinforcement learning and decision-making with the hBayesDM package. Comput Psychiatr. 2017;1:24-57. doi:10.1162/CPSY_a_00002 29601060 PMC5869013

[52] Kruschke JK. What to believe: Bayesian methods for data analysis. Trends Cogn Sci. 2010;14(7):293-300. doi:10.1016/j.tics.2010.05.001 20542462

[53] Gelman A, Tuerlinckx F. Type S error rates for classical and Bayesian single and multiple comparison procedures. Comput Stat. 2000;15(3):373-390. doi:10.1007/s001800000040

[54] Rutledge RB, Moutoussis M, Smittenaar P, . Association of neural and emotional impacts of reward prediction errors with major depression. JAMA Psychiatry. 2017;74(8):790-797. doi:10.1001/jamapsychiatry.2017.1713 28678984 PMC5710549

[55] Dobson KS, Hollon SD, Dimidjian S, . Randomized trial of behavioral activation, cognitive therapy, and antidepressant medication in the prevention of relapse and recurrence in major depression. J Consult Clin Psychol. 2008;76(3):468-477. doi:10.1037/0022-006X.76.3.468 18540740 PMC2648513

[56] Chung D, Kadlec K, Aimone JA, McCurry K, King-Casas B, Chiu PH. Valuation in major depression is intact and stable in a non-learning environment. Sci Rep. 2017;7:44374. doi:10.1038/srep44374 28281665 PMC5345037

[57] Peeters F, Nicolson NA, Berkhof J, Delespaul P, deVries M. Effects of daily events on mood states in major depressive disorder. J Abnorm Psychol. 2003;112(2):203-211. doi:10.1037/0021-843X.112.2.203 12784829

[58] Khazanov GK, Ruscio AM, Swendsen J. The “brightening” effect: reactions to positive events in the daily lives of individuals with major depressive disorder and generalized anxiety disorder. Behav Ther. 2019;50(2):270-284. doi:10.1016/j.ijbiomac.2018.04.150 30824245 PMC6494459

[59] Moutoussis M, Rutledge RB, Prabhu G, . Neural activity and fundamental learning, motivated by monetary loss and reward, are intact in mild to moderate major depressive disorder. PLoS One. 2018;13(8):e0201451. doi:10.1371/journal.pone.0201451 30071076 PMC6072018

[60] Gelman A, Hill J, Yajima M. Why we (usually) don’t have to worry about multiple comparisons. J Res Educ Eff. 2012;5:189-211. doi:10.1080/19345747.2011.618213

