# Supplementary material for: Reinforcement Learning Disruptions in Individuals With Depression and Sensitivity to Symptom Change Following Cognitive Behavioral Therapy
Source: JAMA Psychiatry. 2021 Jul 28;78(10):1–11. doi: 10.1001/jamapsychiatry.2021.1844 (PMC8319827; doi:10.1001/jamapsychiatry.2021.1844)
Supplement: Supplement. — eMethods. eResults. eReferences. eFigure 1. Association of overall performance with MDD diagnosis and symptom severity. eFigure 2. Model fit and parameter recovery. eFigure 3. Posterior distributions and individual means of parameters. eFigure 4. Association of model parameters with model-agnostic summaries of behavior. eFigure 5. Association between anhedonia and reward learning parameters by diagnosis. eFigure 6. Behavioral performance and neural reward signals by depression status and overall depression severity. eFigure 7. Differences in processing of loss outcomes by negative affect. eFigure 8. Stability of parameter estimates over time for control participants. eFigure 9. Parameter changes with time for participants with depression. eFigure10. Diagram of flow of participants through study, including optional CBT portion. eFigure 11. Neural responses associated with symptom improvement. eFigure 12. Schematic depiction of effects of outcome sensitivity and outcome shift on valuation. eTable 1. Reward prediction error, MDD group (n = 69). eTable 2. Reward expected value, MDD group (n = 69). eTable 3. Reward prediction error, controls without depression (n = 32). eTable 4. Reward expected value, controls without depression (n = 32). eTable 5. Loss outcome value correlated with negative affect (MASQ Mixed Distress subscale; n = 101). eTable 6. Loss outcome value, low negative affect participants (n = 52). eTable 7. Loss outcome value, high negative affect participants (n = 49). eTable 8. Depression diagnosis, specifier, severity, medications, and comorbid diagnoses for participants. eTable 9. Exploratory follow-up analyses of associations between symptom change and reinforcement learning parameters in participants with depression who completed CBT. [file jamapsychiatry-e211844-s001.pdf]

Supplemental Online Content

Brown VM, Zhu L, Solway A, et al. Reinforcement learning disruptions in individuals with depression and sensitivity to symptom change following cognitive behavioral therapy. *JAMA Psychiatry*. Published online July 28, 2021. doi:10.1001/jamapsychiatry.2021.1844

eMethods.
eResults.
eReferences.
eFigure 1. Association of overall performance with MDD diagnosis and symptom severity
eFigure 2. Model fit and parameter recovery
eFigure 3. Posterior distributions and individual means of parameters
eFigure 4. Association of model parameters with model-agnostic summaries of behavior
eFigure 5. Association between anhedonia and reward learning parameters by diagnosis
eFigure 6. Behavioral performance and neural reward signals by depression status and overall depression severity
eFigure 7. Differences in processing of loss outcomes by negative affect
eFigure 8. Stability of parameter estimates over time for control participants
eFigure 9. Parameter changes with time for participants with depression
eFigure 10. Diagram of flow of participants through study, including optional CBT portion
eFigure 11. Neural responses associated with symptom improvement
eFigure 12. Schematic depiction of effects of outcome sensitivity and outcome shift on valuation
eTable 1. Reward prediction error, MDD group (n = 69)
eTable 2. Reward expected value, MDD group (n = 69)
eTable 3. Reward prediction error, controls without depression (n = 32)
eTable 4. Reward expected value, controls without depression (n = 32)
eTable 5. Loss outcome value correlated with negative affect (MASQ Mixed Distress subscale; n = 101)
eTable 6. Loss outcome value, low negative affect participants (n = 52)
eTable 7. Loss outcome value, high negative affect participants (n = 49)
eTable 8. Depression diagnosis, specifier, severity, medications, and comorbid diagnoses for participants
eTable 9. Exploratory follow-up analyses of associations between symptom change and reinforcement learning parameters in participants with depression who completed CBT

This supplementary material has been provided by the authors to give readers additional information about their work.

© 2021 Brown VM et al. *JAMA Psychiatry.*

eMethods.

Study design & participants

Inclusion criteria for all participants included: age 18 to 64, English speaking, normal or corrected to normal vision, verbal IQ greater than 80, no contraindications to MRI scanning, no loss of consciousness greater than 30 minutes, no hormonal disorders, no behaviors meeting criteria for substance abuse or dependence (excluding nicotine dependence) in the past 30 days, and no current or past psychotic or bipolar disorders. Clinical and demographic data for participants are reported in Table 1 in the main text. Participants were recruited as part of a larger study assessing neurobehavioral indicators of psychopathology. A total of 2,363 participants made initial contact and were screened for the parent study. Reasons for exclusion included: nonresponse to subsequent contact attempts (N=456), outside age range (N=39), MRI contraindications (N=319), history of loss of consciousness (N=87), current substance abuse or dependence (N=184), mania, psychosis, or subthreshold/remitted depression (N=824), declined participation (N=37), recent treatment (N=93), or, for controls only, poor demographic match to enrolled patients (N=50). Based on a priori power analyses (conducted for effect size of slope = .6 based on prior studies (e.g., Langenecker et al., 2007; Chen et al, 2007), alpha level of .001 to correct for multiple comparisons across the brain volume, and power = 0.9) and expected attrition due to loss to followup and task and fMRI analyses, participants were enrolled until the proposed sample size of 50 per group (no depression, depression receiving CBT, depression not receiving CBT) was met for the larger study. To be included in the present analyses, participants were required to demonstrate engagement on the behavioral task and successfully complete fMRI scanning at study entrance (see Procedures below for further description of exclusion criteria). Sixty-nine participants with depression and 32 controls were included in baseline (i.e., pre-treatment) analyses (total N = 101). Excluded participants did not differ (*p*s > .0.05) from those included at baseline by age; gender; depression diagnosis; severity of depression, anhedonia, or negative affect; education or income level; ethnicity; marital status; or use of psychotropic medications; but had lower estimated IQ as measured by the Wechsler Test of Adult Reading (WTAR)[1].

Participants with a SCID [2] diagnosis of depression were additionally required to have a BDI score greater than 12 on the day of the baseline scan, while nondepressed participants were required to have a BDI score less than 13 [3]. Consistent with previous reports [4,5], symptom severity measures [4,6] were modestly related within participants with depression (R^2 values of .13 to .32) and more strongly related across all participants (R^2 values of .65 to .81), indicating that these measures mapped onto distinct constructs, particularly in participants with clinically elevated symptoms. eTable 8 contains information about depression diagnoses, severity, subtypes, medication classes, and comorbid diagnoses. Possible personality disorders were assessed using the Personality Diagnostic Questionnaire for DSM-IV (PDQ-4)[7], a screener for possible personality pathology, and were not considered exclusionary. Based on the PDQ, 62 participants screened above the cutoff for any personality disorder at baseline (18 for paranoid personality disorder, 0 for histrionic, 4 for antisocial, 31 for obsessive-compulsive, 8 for negativistic, 15 for schizoid, 3 for narcissistic, 31 for avoidant, 31 for depressive, 7 for schizotypal, 5 for borderline, and 12 for dependent personality disorder; note that some participants screened above the cutoff for multiple disorders).

© 2021 Brown VM et al. *JAMA Psychiatry.*

Reinforcement learning task

Participants completed a reinforcement learning task with the goal of maximizing reward and minimizing loss[8–10]. On each trial, participants were presented with two abstract stimuli. One stimulus had a higher (75%) probability of leading to a better monetary outcome and a lower probability (25%) of leading to a worse monetary outcome, while the probabilities for the other stimulus were reversed (i.e., smaller probability of better outcome and larger probability of worse outcome). The participant's choice was framed for a jittered viewing time of 2–4 seconds, after which the outcome (monetary amount gained or lost) was shown for 2 seconds. A fixation cross was shown between each trial for a jittered viewing time of 1–3 seconds. At the beginning of each block, high and low outcome values were randomly chosen with replacement from outcome pairs of {20,70}, {25,75}, or {30,80} and kept consistent within blocks.

An adaptive design titrated task difficulty by ending blocks of trials based on participants' individual learning rather than after a predetermined number of trials. Specifically, block-end criteria included 7 of the last 10 choices being 'correct' (with correct defined as the stimulus more likely to lead to the better outcome); the first block within each valence (reward, loss) was required to be at least 15 trials long. Participants completed an average of 4.03 gain blocks and 4.06 loss blocks and the average number of trials in a block were 14.77 (gain) and 14.49 (loss). The same stimuli were used for all trials within each block, and new stimuli, requiring new learning, were used for each new block. New stimuli were randomly chosen from a stimulus set without replacement for each session; therefore, stimuli were not repeated within session but could be repeated across sessions. On average, a third of the stimuli were re-used between lab visits. Re-use of stimuli was random with respect to participant, symptom severity, diagnostic group, valence (reward vs. loss), and post-treatment symptom improvement (if applicable). Blocks consisted of all reward learning or all loss learning trials and valence (reward, loss) was interleaved across blocks. The task ended when participants completed at least 50 total trials total and at least 25 correct trials per valence. The median number of trials completed for all groups at all timepoints was 50. Severity of anhedonia and negative affect symptoms, as well as presence of a depression diagnosis and severity of depressive symptoms, were all positively correlated ($p < .05$) with completing more gain trials; no symptom or diagnostic measures were correlated with number of loss trials.

Participants completed a practice round prior to entering the scanner. Participants were only instructed that 'one picture is always better than the other'; unknown to them, the structure of the task was such that one stimulus had a 75% chance of leading to the better outcome and a 25% chance of leading to the worse outcome, with opposite probabilities for the other stimulus. Participants were given an initial endowment of $10; final earnings for the learning task were added to payments for the larger battery of biobehavioral measures. To ensure participants were attending to the task and had suitable behavior for model fitting, participants who switched options in either reward or loss blocks less than 5% of the time were excluded from analyses (N=11 [8 with depression and 3 without; comparison of proportion excluded by diagnostic status $\chi^2<0.001$, p=1.0] at baseline and N=2 post-treatment; similar to [11,12]).

Across all participants at baseline, the internal consistency of learning parameters was generally acceptable to excellent (split-half reliability of parameters, median [95% credible interval (CrI)]: gain learning rate 0.955 [0.789:0.998], loss learning rate 0.950 [0.796:0.998], gain outcome sensitivity 0.523 [-0.286:0.938], loss outcome sensitivity 0.790 [-0.352:0.993], gain outcome shift 0.866 [-0.591:0.996], loss outcome shift 0.853 [0.390:0.992]). Internal consistency estimates were similar when examining participants with or without a depression diagnosis separately. Data from nondepressed participants were used to calculate test-retest reliability from baseline to follow-up (n=20; see below for details on participant flow and time intervals). Parameters with sufficiently precise estimates had good test-retest

reliability (median [95% CrI]: gain learning rate 0.703 [-0.345:0.990], loss learning rate 0.775 [0.330:0.977], gain outcome sensitivity 0.787 [-0.120:0.992]; calculated as in[13]). Loss outcome sensitivity [CrI: -0.892:0.978], gain outcome shift [-0.924:0.950] and loss outcome shift [-0.833:0.957] had wide posterior estimates of reliability, indicating insufficient data to move posterior estimates away from uninformative priors (range -1 to 1) due to the small sample size in this analysis. As a result, only medians of parameters estimated with sufficient precision (defined as a 95% CrI range less than 1.5) are reported.

Neuroimaging data collection and preprocessing

Participants were scanned on a 3T Siemens Tim Trio MR scanner. Echoplanar images were collected in 34 4-mm slices at a 30° hyperangulation from the anterior-posterior commissure (AC-PC) line (TR = 2000 ms, TE = 30 ms, flip angle = 90°, matrix = 64 x 64, voxel size = 3.4 x 3.4 x 4.0 mm^3). A high resolution (1 mm^3) anatomical Magnetization Prepared Rapid Gradient Echo (MPRAGE) T1 image (TR = 1200 ms, TE = 2.66 ms, flip angle = 12°) was collected to aid in registration.

Preprocessing and all further imaging analyses were conducted using SPM8 for fMRI (Wellcome Trust Centre for Neuroimaging, http://www.fil.ion.ucl.ac.uk/spm/software/spm8/) and consisted of slice timing correction, realignment to the first functional image, coregistration to the participant's high-resolution structural image, normalization to the MNI template, and smoothing to ensure Gaussianity (6mm FWHM). Participants with motion greater than 3 mm or 0.05 radians in any direction or who had incomplete scanning data were excluded. The proportion of participants excluded for motion or incomplete scanning data did not differ for participants with versus without depression at baseline or post-treatment timepoints (all $\chi^2<3.2$, all *p*s>0.05).

Cognitive-behavioral therapy

After completing baseline study procedures, participants with depression were offered 12 weeks of cognitive-behavioral therapy. The naturalistic design of our study meant that participants with depression were free to enroll in the treatment phase of the study or to decline treatment. Patients receiving treatment were treated by experienced licensed psychologists (25.6 +/- 8.6 years in practice) using the manual by Munoz & Miranda [14]. Participants could complete up to 12 weekly sessions of therapy but were considered to have an adequate course of CBT if they completed at least eight sessions; most participants (25/28) completed twelve sessions. After 12 weeks, participants completed allstudy procedures again; depressed and nondepressed participants did not differ on time between assessments (mean [SD] number of days between first and second time point: patients 115 [2.8], controls 111 [2.3], $t_{57} = 0.930$, $p > .1$). Twenty-eight depressed participants and 20 controls had suitable data at both time points for analyses of pre- to post-treatment behavioral and neural changes (see eFigure 8 for a diagram of participant flow through treatment). Participants with depression who completed follow-up assessments but who had declined (n=8) or did not complete (n=3) treatment are investigated in exploratory follow-up analyses (below); these participants were not included in primary analyses due to concerns about systematic differences due to non-random assignment. Similar to baseline analyses, treatment completers did not differ from patients who did not complete treatment or who were excluded from analyses on any clinical or demographic measures except estimated IQ. Participants who were lost to follow-up, regardless of treatment selection, did not differ from those who

on any clinical or demographic measure, both across all participants and within participants with a depression diagnosis only.

Model-agnostic analysis of behavior at baseline

Model-agnostic analyses assessed the relationship between non-model-based and model-based analyses and between overall performance and symptom severity.

To broadly examine the relationships between model-derived learning parameters and model-agnostic measures of behavior, individual estimates for mean values of each learning parameter, separated by reward and loss learning, were extracted and compared to summary behavioral measures. These analyses confirmed expected relationships between these model-based and model-agnostic measures (see eFigure 4 for plots of individuals' model-derived mean learning parameter values against individuals' proportions of correct choices, representing overall performance on the task, and proportion of trials with switches between options, representing less consistency in choices). We note the unique relationships of each model-derived learning parameter with these traditional summary variables; these relationships illustrate how differences in components of learning patterns, indexed by combinations of increases and decreases in model-derived parameter values, may be obscured when viewing summary variables only.

Learning patterns independent of a formal computational model were investigated by assessing the relationship between model-agnostic learning measures and baseline symptoms, with performance defined as the proportion of choices options more likely to lead to higher outcomes. Reward and loss performance were negatively but nonsignificantly related to all symptoms (eFigure 1; all ps > 0.05), except for a positive but non-significant relationship between reward performance and anhedonia in participants with depression (t_{65} = 1.18, p = .2).

Model estimation

Participants' choices were fit to models using hierarchical Bayesian estimation, which estimated the distribution of each free parameter over the group of participants and for each participant individually [15–17]. By allowing group and individual level distributions over parameters and allowing each level to inform estimates at other levels, hierarchical Bayesian estimation more accurately recovers true parameters, especially parameters that are somewhat correlated as is often the case in reinforcement learning models [18–20]. Posterior distributions were estimated using Hamiltonian Monte Carlo with a No-U-Turn Sampler (HMC with NUTS) as implemented in Stan via its RStan interface ([21], version 2.19). Group level parameters were specified as normally distributed, with a lower bound of 0 on outcome sensitivity (or inverse temperature, in models with this parameter) to constrain the parameter to be positive. Parameters were given a non-centered parameterization to aid in estimation by specifying mean, scale, and error distributions for each parameter [22]. Similar to [23,24], mean distributions, estimated at the group level, were specified as normally distributed with priors of mean = 0 and standard deviation = 10, for parameters that were not logit transformed, or with standard deviation = 2.5 for parameters that were logit transformed. Scale distributions, estimated at the group level, were given a half-Cauchy prior [25] (bounded to be greater than 0) with values of 0 and 2.5 (0 and 2 for parameters that were logit transformed). Error distributions, which were estimated for each subject, were given a normal prior with mean = 0 and standard deviation = 1. Similarly, effects of covariates, and for treatment analyses, effects of time, treatment, and interactions were given a normal prior with mean = 0 and standard deviation = 1.

© 2021 Brown VM et al. *JAMA Psychiatry.*

For learning rate, these parameter values were then run through a logistic transformation to bound values between 0 and 1. Therefore, each subject's parameter (for example, learning rate) consisted of a group estimated mean value plus the combined value of the group estimated scale value multiplied by the individually estimated error value. The effects of covariates were assumed to adjust the mean of each parameter (due to homogeneity of variance), and so acted on the mean value of the parameter per subject. Models were estimated separately for reward and loss learning except where noted. Four chains were run for each valence, with 8000 samples per chain (4000 after discarding warm-up samples). Chains were visually inspected for convergence and showed good mixing, with all values of the potential scale reduction factor [26] less than 1.1.

Reinforcement learning model specification and validation

Model selection. Model-based analyses were conducted using reinforcement learning models [27,28]. Model parameters were based on previous work on learning differences in depression and differential learning from rewards and losses [20,29–31], with a particular focus on parameters assessing differences in updating versus valuation. Updating was represented by a learning rate parameter α, indexing how rapidly the expected value *Q* was updated based on prediction error, which is the difference between received value *R* and expected value *Q*:

$$Q_{t+1} = Q_t + \alpha * (R'_t - Q_t)$$

Two possible explanations for altered valuation were considered: first, a difference in relative scaling known as outcome sensitivity (ρ; similar to [20]): $R'_t = \rho * R_t$, (where *R'* indicates an altered valuation of received value *R*), or an overall shift (τ) in valuation of outcomes: $R'_t = \tau + R$. This second possible change in valuation, by shifting all values, also allows for valence-sensitive rescaling of values often seen when learning from rewards versus losses [30]. To differentiate the effects of outcome sensitivity and outcome shift, outcome sensitivity was multiplied on the more extreme outcome only (+/-$0.70 to $0.80).

In addition, learning could be affected not by changes in valuation, but by changes in decision processes. These decision effects can be represented by inverse temperature β (an exploration/exploitation parameter which functions similarly to outcome sensitivity ρ but acts at the choice and not valuation stage):

$$P(A)_t = \frac{e^{\beta * Q(A)_t}}{(e^{\beta * Q(A)_t} + e^{\beta * Q(B)_t})}$$

Where $P(A)_t$ represents the probability of choosing option A at trial *t*. Decision effects can also be represented by choice perseveration ω (which functions similarly to outcome shift τ but acts at the choice rather than valuation stage [32]):

$$P(A)_t = \frac{e^{\beta * Q(A)_t + choice_{t-1} * \omega}}{(e^{\beta * Q(A)_t + choice_{t-1} * \omega} + e^{\beta * Q(B)_t + |(1 - choice_{t-1})| * \omega})}$$

where $choice_{t-1}$ is 1 if the stimulus (A in this example) was chosen on the previous trial and 0 if it was not.

To test which combination of parameters best represented participants' behavior on the task, models with learning rate α plus 1) one valuation parameter, outcome sensitivity ρ (similar to [20]; model $\alpha + \rho$; 2 free parameters for reward and loss learning, respectively); 2) both valuation parameters (model $\alpha + \rho + \tau$; 3 free parameters per valence); 3) one decision parameter, inverse temperature β (model $\alpha + \beta$; 2 free parameters per valence); 4) both decision parameters (model $\alpha + \beta + \omega$; 3 free parameters per valence) were tested. Models without inverse temperature β as a free parameter fixed this parameter based on its estimated value ($\beta \approx 7$).

Model fit was compared with integrated BIC (iBIC) computed from the posterior distribution, penalizing for the number of parameters. A lower iBIC indicates a better fitting model [33]. Model fit was tested with models fit across all participants, within participants with depression only, and within participants without depression only to ensure the winning model was the same regardless of diagnosis. Using a model fit measure based on BIC, rather than other measures such as AIC, penalizes models with extra parameters more severely and is more conservative about overfitting data to models with excessive parameters [34]; a corollary of this effect is that if a BIC-based measure selects a more complex model, an AIC-based measure is guaranteed to select the same model. Integrated BIC also accounts for the hierarchical, dependent nature of individuals' model fits which is not possible with indices requiring individual fit statistics (e.g., Bayesian model selection; [35]). The best fitting model (lowest iBIC) for both reward and loss learning included learning rate, outcome sensitivity, and outcome shift (model $\alpha + \rho + \tau$; eFigure 2). We further confirmed the role of the outcome shift parameter by examining the relationship between relative model fits of models with and without this parameter and proportion of behavior switches by participant and valence. Since the outcome shift parameter changes the value of the currently chosen option, making the currently chosen option more or less likely to be chosen in the future, adding this parameter should improve the fit for people who switch less than average. We found this effect, and particularly during loss learning (regression of proportion of behavioral switches on difference in per-trial log likelihood between a model with versus without outcome shift: gain $t_{99} = -1.926$, $p=0.057$; loss $t_{99} = -9.388$, $p<.001$).

Independence of model parameters. Reinforcement learning model parameters can show collinearity between parameters assessing value updates (such as learning rate) and value scaling (such as inverse temperature or outcome sensitivity) [15,20,24]. Therefore, parameter recovery, correlation of individual parameter estimates, inspection of posterior distributions, and analysis of relationships between parameters and model-agnostic behavioral summaries were carried out to ensure parameters were independently estimated and related to behavior. First, model recovery was performed for different combinations of parameter values. Specifically, simulated data for 100 subjects was created with combinations of three different levels of the mean value of each parameter (.25, .50, and .75 for reward and loss learning rate; .50, 1.0, and 1.5 for reward and loss outcome sensitivity; -.50, -.25, and 0 for reward outcome shift; and 0, .50, and 1.0 for loss outcome shift), resulting in 27 overall sets of simulated data with different combinations of parameters. Values were chosen based on the range of parameters in real participants' behavior. Model parameters were estimated for this simulated data and recovered parameter values were plotted against simulated parameter values to verify parameters could be independently estimated at different values. Importantly, this validation ensured similar values could be

recovered from one simulated value of a parameter while varying the simulated values of all other parameters.

Next, using empirical data from all participants estimated as one group, individual estimates were extracted for each parameter and correlated. No parameters within a valence (reward, loss learning) showed correlations above 0.4. Then, to ensure parameters were independently identifiable, the samples from the posterior distributions for each parameter were plotted against the other parameters in each valence to allow visual inspection of any correlations or trade-offs in the value of each parameter across its posterior distribution. Lastly, relationships between each parameter and model-agnostic behavioral summaries (proportion of correct choices and switches) were calculated to determine whether parameters showed differential relationships with overall behavior on the task.

All model validation methods including parameter recovery (eFigure 2A), inspection of posterior probability distributions (eFigure 3A), correlation of empirical individual parameter estimates (eFigure 3B), and relationships with model-agnostic behavioral summaries (eFigure 4) indicated that the parameters of the best fitting model were recoverable and uniquely identifiable. Of note, whether parameters can be independently estimated in reinforcement learning models is affected by a number of factors, including the model employed, the task performed by participants, and the amount of data collected at subject and group levels, but is particularly affected by the method used to estimate parameters. The fully Bayesian hierarchical estimation implemented by the HMC NUTS algorithm used here explores posterior distributions in a manner that greatly reduces intercorrelations among parameters and improves recovery of simulated parameters [22], resulting in an improved ability to estimate uncorrelated parameters compared to other work with similar models. As shown in eFigure 3B, the correlations between learning rate and outcome sensitivity were in opposite directions by valence (positive correlation for reward and negative for loss). This difference is due to the differential relationship between these parameters by valence - in reward learning, the outcome sensitivity parameter is multiplied on the higher reward, and participants are learning to achieve this reward. For loss trials, this parameter is multiplied on the higher loss, and participants learn to avoid this loss, leading to mirrored effects on parameter tradeoffs between reward and loss learning.

<u>Differentiation of learning by valence (reward, loss).</u> Based on previous work, we assumed a priori that learning patterns differed for rewards and losses [31,36,37]. Nonetheless, to test whether participants' behavior was better characterized by combining or separating parameters across reward and loss blocks, models with (1) all parameters combined across reward and loss (total number of parameters = 3), (2) each parameter split between valence in turn (e.g., learning rate split between valence while outcome sensitivity and outcome shift were combined, repeated in turn for outcome sensitivity and outcome shift; total number of parameters per model = 4), and (3) all parameters split between valence (total number of parameters = 6). The integrated BIC (iBIC) was again used to measure model fit, with lower iBIC indicating a better fit. To check if the best fitting model was the same across groups, iBIC was calculated across all participants as well as within groups of participants with and without depression separately. Including unique parameters for reward and loss learning improved model fit over combining some or all parameters across valence (eFigure 2c); this provided support for the posited valence- sensitive learning processes.

Assessment of model based behavioral differences at baseline

Estimation of parameters using both group and individual level information may introduce dependencies among the individual level estimates, in that using participants' parameters on an individual basis to compare against outside measures (e.g., symptom severity, diagnosis) can be biased [25,38,39]. Therefore, to examine relationships between parameters and variables of interest, the effects were estimated within the model by introducing another parameter to index the effect of the variable of interest [39,40]. To do so, covariates were z-scored or, in the case of binary variables, dummy coded, and entered into a regression during model estimation to predict the mean of a parameter. For example, the following analysis determines the effect of anhedonia on learning rate:

$$\alpha_{total} = \alpha_{intercept} + \text{anhedonia}*\alpha_{anhedonia} + \varepsilon$$

In this manner, $\alpha_{anhedonia}$ represents the effect of (standardized) anhedonia severity on learning rate. To determine significance, 95% of the posterior distribution of these parameters was required to not include0 (i.e., to be entirely above or below 0 [41]). To assess whether results were unique to one primary subscale of the MASQ (anhedonia, negative affect, or arousal) relative to the others at baseline and to reduce the number of independent tests, analyses with all three of these measures were also run in the same model. To account for potential nonlinearities in the relationship between symptoms and behavior across clinical and non-clinical levels of depression, models were also run within the depressed participants only as well as across all participants (total number of independent tests: 2 for reward learning (all participants and depressed participants only) and 2 for loss learning). To control the total probability of concluding that a learning-symptom relationship existed in a direction that it did not ('Type S error'), we restricted experimentwise Type S error to 5%[42]. Note that as a scaling parameter, learning rate lies only in the range of 0 to 1, but to aid in estimation, this parameter was estimated as a continuous variable and then logistically transformed to be bounded between 0 and 1. Therefore, to ease interpretation the effects of symptoms on learning rate were reported for transformed (range of 0 to 1) values. Analyses were also run with estimated IQ and presence of psychotropic medication as additional covariates, but inclusion of these covariates did not meaningfully change any results.

Power analyses

To determine what effect sizes we were powered to detect, we simulated 100 datasets with 80% power to detect effects where they exist. We measured power as the percent of simulated datasets where 95% of the posterior distribution of the relationship between symptom severity and learning parameters was significantly more or less than 0. Posterior distributions of effect sizes for regression coefficients (f^2) were then computed from these simulated datasets using Bayesian R^2 [43]. Effect sizes were computed for sample sizes for all participants at baseline (N=101) and for participants with depression only (N=69) and were interpreted according to [44], where $f^2 > 0.02$ is a small effect size, $f^2 > 0.15$ medium, and $f^2 > 0.35$ large. For N=101, we were powered to detect small effect sizes (median $f^2 = 0.106$) and for N=69, we were powered to detect medium effect sizes (median $f^2 = 0.326$).

Baseline imaging analyses

First level imaging analyses used parametric regressors of prediction error δ or outcome value R_t at the time of outcome and expected value Q_t of the chosen option at the time of onset. Prediction error and expected value were calculated based on participants' individually estimated parameters from the reinforcement learning model and were z-transformed prior to entering in the imaging model [45]. Regressors were separated by valence (reward or loss) and all regressors were modeled as stick

© 2021 Brown VM et al. *JAMA Psychiatry.*

functions. Additional regressors of no interest were included for button presses, block number, and six motion parameters. Data were high pass filtered with a cutoff of 128 seconds.

Primary group level analyses focused on ventral striatum and ventromedial prefrontal cortex, brain areas known to be central to reinforcement learning [46,47]. Regions of interest were defined from the peak coordinates from a meta-analysis of prediction error and expected value BOLD response in reinforcement learning tasks [48]; specifically, a 6 mm sphere was drawn around the peak coordinate from right and left striatum from prediction error-related activation and subgenual anterior cingulate cortex from expected value-related activation. The first eigenvariate of the beta values in each ROI from prediction error, outcome value, and expected value activations was extracted for each participant and regressed against measures of interest.

Additional whole brain analyses were run to examine contributions of expected value and outcome value signals at the outcome time point. Whole brain imaging analyses used an initial cluster defining threshold of $p < .001$ and a cluster-level topological FDR significance of $p < .05$ [49].

To relate neural activation to symptom measures, BOLD activity (ROI values and whole-brain activation) were correlated with symptom measures. The relationship between expected value and prediction error related activity and symptom measures was further tested by testing the interaction of prediction error neural signal and symptom measures on expected value neural signal in striatal ROIs (as in [50]). Analyses were also run with estimated IQ and presence of psychotropic medication as additional covariates; inclusion of these covariates did not meaningfully change any results.

Given the effect of negative affect on prediction error signaling in subgenual ACC, exploratory follow up analyses were performed. Specifically, as prefrontal cortical signals in areas like subgenual anterior cingulate cortex are linked more to value representation than prediction error itself[51,52], we examined thetwo value-related components of prediction error (i.e., ‘expected’ value and ‘actual’ value received) to determine if either explained the relationship between reduced prediction error signaling in subgenual anterior cingulate and negative affect in a way that reflected the model-based behavioral findings of a more negative outcome shift parameter. This analysis revealed that greater negative affect was negatively related to (that is, less modulated by) signaling of ‘actual’ outcome value in ventromedial prefrontal cortex and precuneus ($p < .05$ corrected; eFigure 6a; eTable 5), with no significant relationship with ‘expected value’ either at time of cue onset or outcome; outcome shift parameter values did not mediate this relationship (see eFigure 6b and Tables S6 and S7 for neural processing of outcome value within participants low and high in negative affect separately). This result suggests that participants greater in negative affect differ in neural processing of actual outcome values, with subsequent effects on prediction error, rather than in processing expected value.

Behavioral analyses of symptom change and symptom-independent change over time

To investigate the relationship between changes in reinforcement learning parameter values and changes in symptoms with the depressed participants, analyses were run assessing the interaction of changes in symptoms with time on learning parameters (2 independent statistical tests: 1 each for reward and loss learning). Changes in symptoms were defined as post- minus pre-treatment, such that higher values indicated more improvement. Since the learning variables of interest were assessed at pre- and post-treatment only, including this slope as well as baseline severity in analyses is analogous to an mixed effects model[53] assessing changes across time within each person and relating these changes to

symptoms across people. Similar to baseline analyses, the mean of each parameter was estimated as a regression of the intercept of the parameter plus main effects and interactions of time and symptoms. Specifically, analyses included the within-subject main effect of time (dummy coded for 0 = first session and 1 = second session), the main effect of baseline symptoms, and the interaction of time and symptoms[54]:

$$\alpha_{total} = \alpha_{intercept} + time*\alpha_{time} + baseline\_anhedonia*\alpha_{anhedonia} + time*\Delta anhedonia*\alpha_{time*symptom} + \varepsilon$$

In this analysis, the $\alpha_{symptom*time}$ parameter assesses the change in learning rate from the first to second session related to improvement (slope) in anhedonia, while the α_{time} parameter assesses the change in learning rate across sessions unrelated to improvement in anhedonia. The parameters for intercept, time, and time*symptom were allowed to vary by participant (i.e., an uncorrelated random intercept and random slope model [55] for analyses with total symptom change; for analyses with individual subscales, random slope models led to a lack of convergence and so only random intercept models were used). Analyses were also run with estimated IQ, presence of psychotropic medication, and depression specifiers (melancholic depression, atypical depression) as additional time-independent covariates in separate analyses; inclusion of these covariates did not meaningfully change any results.

Exploratory analyses of CBT-specific effects

To assess whether relationships between changes in learning and symptoms were specific to symptom improvement after CBT, participants who completed treatment were compared to a small group ('non-CBT') of participants who were depressed at baseline but declined (n=8) or did not complete treatment (n=2: both attended only three sessions) but returned for follow-up assessment. Analyses compared the 28 participants who completed treatment to the 10 who declined or did not complete treatment. One additional participant enrolled in CBT but did not complete an adequate number of sessions (sessions = 5) and so was excluded from the CBT vs. no-CBT comparison. CBT depressed, non-CBT depressed, and nondepressed participants did not differ on time between assessments (mean [SD] number of days between first and second time point: CBT depressed 117 [18.2], non-CBT depressed 110 [12.9], and nondepressed 111 [10.1], $F_{1,57} = 0.01$, $p > .1$).

To investigate differences in learning parameters between the CBT and non-CBT group, model-based analysis included an additional main effect and interactions with treatment:

$$\alpha_{total} = \alpha_{intercept} + time*\alpha_{time} + baseline\_anhedonia*\alpha_{anhedonia} + treatment*\alpha_{treatment} + time*\Delta anhedonia*\alpha_{time*symptom} + time*treatment*\alpha_{time*treatment} + baseline\_anhedonia*treatment*\alpha_{symptom*treatment} + \Delta anhedonia*treatment*time*\alpha_{symptom*time*treatment} + \varepsilon$$

In this analysis, the $\alpha_{symptom*time*treatment}$ parameter assesses the change in learning rate from the first to second session in the CBT relative to no-CBT group related to improvement (slope) in anhedonia, while the $\alpha_{symptom*time}$ parameter assesses the change in learning rate from the first to second session *independent* of treatment condition.

© 2021 Brown VM et al. *JAMA Psychiatry.*

eResults

Baseline results: depression diagnosis and severity

Reward learning. No parameters significantly differed by depression diagnosis (learning rate: mean = 0.041 (standardized mean = 0.348), CrI -0.077:0.265; outcome sensitivity: -0.233 (1.70), CrI 0.512:0.028;outcome shift: 0.132 (1.77), CrI -0.013:0.283) or by depression severity (BDI; learning rate: -0.002 (- 0.033), CrI -0.074:0.127; outcome sensitivity: -0.084 (-1.40), CrI -0.205:0.033; outcome shift: 0.045 (1.32), CrI -0.022:0.111).

Loss learning. No parameters significantly differed by depression diagnosis (learning rate: 0.074 (0.736), CrI -0.050:0.292; outcome sensitivity: 0.395 (1.08), CrI -0.346:1.09; outcome shift: -0.147 (-1.44), CrI -0.356:0.046) or by depression severity (learning rate: 0.055 (1.06), CrI -0.030:0.170; outcome sensitivity: 0.312 (1.62); outcome shift: -0.072 (-1.48), CrI -0.170:0.020).

Baseline results: simultaneous analysis of three MASQ subscales

Results were similar when assessing all MASQ scales in the same analysis compared to separate analyses: for reward learning in participants with depression, anhedonia was related to higher learningrate and lower outcome sensitivity (learning rate: mean = -0.120 (standardized mean = -2.24), CrI 0.106:-0.005; outcome sensitivity: 0.270 (2.41), CrI 0.063:0.505) . For loss learning in all participants, negative affect was marginally related to lower outcome shift (mean = -0.178 (standardized mean = 1.65), CrI -0.392:0.031). No additional significant associations between symptoms and learning parameters emerged when examining all three scales together.

Analyses of parameter change: symptom-independent changes over time

Independent of changes in symptoms, participants with depression showed increased reward outcome sensitivity (mean change = 1.14 (standardized mean change = 2.77), CrI 0.434:2.04), decreased reward outcome shift (-0.349 (-2.54) CrI -0.107:-0.649), decreased loss learning rate (-0.167 (-1.75) CrI - 0.054:-0.156), and decreased loss outcome sensitivity (-1.73 (-2.77) CrI -0.537:-2.98). Reward learning rate (- 0.180 (-1.45), CrI -0.155:0.02) and loss outcome shift (0.253 (1.58) CrI -0.055:0.577) did not significantly change across visits. Overall shifts in parameters are shown in eFigure 9A and estimates ofparameters of participants with depression at each time point are shown in eFigure 9B. Testing changesover time independent of symptoms showed the same pattern of effects, confirming that symptom- related and symptom-independent changes with time could be independently estimated.

Analyses of symptom change: exploratory CBT-specific effects

Exploratory analyses compared participants electing to receive CBT to those who did not, focusing on parameters showing changes with symptoms during treatment (gain learning rate and loss outcome shift). During gain learning, learning rate showed a trending CBT-specific increase with symptom change (learning rate: mean = 0.171 and CrI = -0.001 to 0.018), with no relationship between changes in learning and symptom change independent of treatment condition (-0.005, CrI = -0.022 to 0.239). Duringloss learning, outcome shift showed nonsignificant increases with symptom change that were of similar magnitude for effects specific to (0.345, CrI = -0.418:1.14) and independent (mean = 0.233 and CrI = -0.323 to 0.817) of CBT. No other parameters during gain or loss learning showed significant or trending relationships with symptom change.

© 2021 Brown VM et al. *JAMA Psychiatry.*

eReferences

1. Wechsler D. *Wechsler Test of Adult Reading: WTAR*. Psychological Corporation; 2001.

2. First MB, Spitzer R, Gibbon M, Wiliams J. *User's Guide for the Structured Interview for DSM-IV Axis I Disorders—Research Version (SCID-I)*. Biometrics Research; 1996.

3. Kendall PC, Hollon SD, Beck AT, Hammen CL, Ingram RE. Issues and recommendations regarding use of the Beck Depression Inventory. *Cognit Ther Res*. 1987;11(3):289-299.

4. Watson D, Weber K, Assenheimer JS, Clark LA, Strauss ME, McCormick RA. Testing a tripartite model: I. Evaluating the convergent and discriminant validity of anxiety and depression symptom scales. *J Abnorm Psychol*. 1995;104(1):3-14. http://www.ncbi.nlm.nih.gov/pubmed/7897050

5. Buckby JA, Yung AR, Cosgrave EM, Killackey EJ. Clinical utility of the Mood and Anxiety Symptom Questionnaire (MASQ) in a sample of young help-seekers. *BMC Psychiatry*. 2007;7(1):50. doi:10.1186/1471-244X-7-50

6. Steer RA, Ball R, Ranieri WF, Beck AT. Dimensions of the Beck Depression Inventory-II in clinically depressed outpatients. *J Clin Psychol*. 1999;55(1):117-128. http://www.ncbi.nlm.nih.gov/pubmed/10100838

7. Hyler SE. *Personality Questionnaire (PDQ-4+)*. New York State Psychiatric Institute; 1994.

8. Pessiglione M, Seymour B, Flandin G, Dolan RJ, Frith CD. Dopamine-dependent prediction errors underpin reward-seeking behaviour in humans. *Nature*. 2006;442(7106):1042-1045. doi:10.1038/nature05051

9. Brown VM, Zhu L, Wang JM, Frueh BC, King-Casas B, Chiu PH. Associability-modulated loss learning is increased in posttraumatic stress disorder. *Elife*. 2018;7:e30150. doi:10.7554/eLife.30150

10. Wang JM, Zhu L, Brown VM, et al. In Cocaine Dependence, Neural Prediction Errors During Loss Avoidance Are Increased With Cocaine Deprivation and Predict Drug Use. *Biol Psychiatry Cogn Neurosci Neuroimaging*. 2019;4(3). doi:10.1016/j.bpsc.2018.07.009

11. Klein-Flügge MC, Kennerley SW, Saraiva AC, Penny WD, Bestmann S. Behavioral modeling of human choices reveals dissociable effects of physical effort and temporal delay on reward devaluation. Torres-Oviedo G, ed. *PLOS Comput Biol*. 2015;11(3):e1004116. doi:10.1371/journal.pcbi.1004116

12. Sokol-Hessner P, Hsu M, Curley NG, Delgado MR, Camerer CF, Phelps E a. Thinking like a trader selectively reduces individuals' loss aversion. *Proc Natl Acad Sci U S A*.

© 2021 Brown VM et al. *JAMA Psychiatry.*

2009;106(13):5035-5040. doi:10.1073/pnas.0806761106

13. Brown VM, Chen J, Gillan CM, Price RB. Improving the reliability of computational analyses: Model-based planning and its relationship with compulsivity. *Biol Psychiatry Cogn Neurosci Neuroimaging*. Published online 2020. doi:10.1016/j.bpsc.2019.12.019

14. Munoz RF, Miranda J. *Individual Therapy Manual for Cognitive-Behavioral Treatment for Depression*. RAND; 1996.

15. Daw ND. Trial-by-trial data analysis using computational models. In: Delgado MR, Phelps EA, Robbins TW, eds. *Decision Making, Affect, and Learning: Attention and Performance XXIII*. ; 2011:3-38.

16. Wiecki T V, Poland J, Frank MJ. Model-based cognitive neuroscience approaches to computational psychiatry: Clustering and classification. *Clin Psychol Sci*. 2015;3(3):378-399.

17. Ahn W-Y, Haines N, Zhang L. Revealing neuro-computational mechanisms of reinforcement learning and decision-making with the hBayesDM package. *Comput Psychiatry*. 2017;1:24-57. doi:10.1101/064287

18. Ahn W-Y, Krawitz A, Kim W, Busemeyer JR, Brown JW. A model-based fMRI analysis with hierarchical Bayesian parameter estimation. *J Neurosci Psychol Econ*. 2011;4(2):95-110. doi:10.1037/a0020684.A

19. Gershman SJ. Empirical Priors for Reinforcement Learning Models. *J Math Psychol*. 2016;71:1-6. doi:10.1016/j.jmp.2016.01.006

20. Huys QJ, Pizzagalli DA, Bogdan R, Dayan P. Mapping anhedonia onto reinforcement learning: A behavioural meta-analysis. *Biol Mood Anxiety Disord*. 2013;20(1):1-29. doi:10.1186/2045-5380-3-12

21. Carpenter B, Gelman A, Hoffman M, et al. Journal of Statistical Software Stan : A Probabilistic Programming Language. *J Stat Softw*. 2016;VV(Ii).

22. Betancourt M, Girolami M. Hamiltonian Monte Carlo for Hierarchical Models. In: *Current Trends in Bayesian Methodology with Applications*. ; 2015:79-101.

23. Gillan CM, Otto AR, Phelps EA, Daw ND. Model-based learning protects against forming habits. *Cogn Affect Behav Neurosci*. 2015;(March):523-536. doi:10.3758/s13415-015-0347-6

24. Otto AR, Raio CM, Chiang A, Phelps EA, Daw ND. Working-memory capacity protects model-based learning from stress. *Proc Natl Acad Sci*. 2013;110(52):20941-20946. doi:10.1073/pnas.1312011110

25. Gelman A, Carlin JB, Stern HS, Dunson DB, Vehtari A, Rubin DB. *Bayesian Data Analysis*. Third. CRC Press; 2014.

26. Gelman A, Rubin DB. Inference from iterative simulation using multiple sequences. *Stat Sci*.

© 2021 Brown VM et al. *JAMA Psychiatry.*

1992;7(4):457-511. doi:10.1214/ss/1177011136

27. Rescorla RA, Wagner AR. A theory of Pavlovian conditioning: Variations in the effectiveness of reinforcement and nonreinforcement. In: *Classical Conditioning II: Current Research and Theory*. ; 1972:64-99.

28. Sutton RS, Barto AG. *Reinforcement Learning: An Introduction*. MIT Press; 1998.

29. Rothkirch M, Tonn J, Kohler S, Sterzer P. Neural mechanisms of reinforcement learning in unmedicated patients with major depressive disorder. *Cereb Cortex*. Published online 2017:1-11. doi:10.1093/cercor/bhw393

30. Palminteri S, Khamassi M, Joffily M, Coricelli G. Contextual modulation of value signals in reward and punishment learning. *Nat Commun*. 2015;6:8096. doi:10.1038/ncomms9096

31. Pessiglione M, Seymour B, Flandin G, Dolan RJ, Frith CD. Dopamine-dependent prediction errors underpin reward-seeking behaviour in humans. *Nature*. 2006;442(7106):1042-1045. doi:10.1038/nature05051

32. Rutledge RB, Lazzaro SC, Lau B, Myers CE, Gluck MA, Glimcher PW. Dopaminergic drugs modulate learning rates and perseveration in Parkinson's patients in a dynamic foraging task. *J Neurosci*. 2009;29(48):15104-15114. doi:10.1523/JNEUROSCI.3524-09.2009

33. Guitart-Masip M, Huys QJM, Fuentemilla LL, Dayan P, Duzel E, Dolan RJ. Go and no-go learning in reward and punishment: Interactions between affect and effect. *Neuroimage*. 2012;62(1):154-166. doi:10.1016/j.neuroimage.2012.04.024

34. Burnham K, Anderson D. *Model Selection and Multimodel Inference: A Practical Information-Theoretic Approach*. (Springer, ed.).; 2002.

35. Rigoux L, Stephan KE, Friston KJ, Daunizeau J. Bayesian model selection for group studies - Revisited. *Neuroimage*. 2014;84(4):971-985. doi:10.1016/j.neuroimage.2013.08.065

36. Harrison NA, Voon V, Cercignani M, Cooper EA, Pessiglione M, Critchley HD. A neurocomputational account of how inflammation enhances sensitivity to punishments versus rewards. *Biol Psychiatry*. 2016;80(1):73-81. doi:10.1016/j.biopsych.2015.07.018

37. Brown VM, Zhu L, Wang JM, Frueh C, King-Casas B, Chiu PH. Associability-modulated loss learning is increased in posttraumatic stress disorder. *Elife*. 2018;7:e30150. doi:10.7554/eLife.30150

38. Efron B, Morris C. Stein's paradox in statistics. *Sci Am*. 1977;236:119-127.

39. Boehm U, Steingroever H, Wagenmakers E-J. Using Bayesian regression to test hypotheses about relationships between parameters and covariates in cognitive models. *Behav Res Methods*. Published online 2017. doi:10.3758/s13428-017-0940-4

40. Cavanagh JF, Wiecki T V, Kochar A, Frank MJ. Eye tracking and pupillometry are indicators of

© 2021 Brown VM et al. *JAMA Psychiatry.*

dissociable latent decision processes. *J Exp Psychol Gen*. 2014;143(4):1476-1488. doi:10.1037/a0035813

41. Kruschke JK, Liddell TM. The Bayesian New Statistics: Hypothesis testing, estimation,meta-analysis, and power analysis from a Bayesian perspective. *Psychon Bull Rev*. 2018;25(1):178-206. doi:10.3758/s13423-016-1221-4

42. Gelman A, Tuerlinckx F. Type S error rates for classical and Bayesian single and multiple comparison procedures. *Comput Stat*. 2000;15(3):373-390. doi:10.1007/s001800000040

43. Gelman A, Goodrich B, Gabry J, Vehtari A. R-squared for Bayesian Regression Models. *Am Stat*.2019;73(3):307-309. doi:10.1080/00031305.2018.1549100

44. Cohen J. *Statistical Power Analysis for the Behavioral Sciences*. Second. Lawrence Erlbaum Associates; 1988.

45. Lebreton M, Palminteri S. Assessing inter-individual variability in brain-behavior relationship with functional neuroimaging. *bioRxiv*. Published online 2016:1-14.

46. Rangel A, Camerer CF, Montague PR. A framework for studying the neurobiology of value-based decision making. *Nat Rev Neurosci*. 2008;9(7):545-556. doi:10.1038/nrn2357

47. O'Doherty JP, Hampton AN, Kim H. Model-based fMRI and its application to reward learning and decision making. *Ann N Y Acad Sci*. 2007;1104:35-53. doi:10.1196/annals.1390.022

48. Chase HW, Kumar P, Eickhoff SB, Dombrovski AY. Reinforcement learning models and their neural correlates: An activation likelihood estimation meta-analysis. *Cogn Affect Behav Neurosci*. Published online February 10, 2015. doi:10.3758/s13415-015-0338-7

49. Woo CW, Krishnan A, Wager TD. Cluster-extent based thresholding in fMRI analyses: Pitfalls and recommendations. *Neuroimage*. 2014;91:412-419. doi:10.1016/j.neuroimage.2013.12.058

50. Greenberg T, Chase HW, Almeida JR, et al. Moderation of the relationship between reward expectancy and prediction error-related ventral striatal reactivity by anhedonia in unmedicated major depressive disorder: Findings from the EMBARC study. *Am J Psychiatry*. 2015;172(9):881-891. doi:10.1176/appi.ajp.2015.14050594

51. Gläscher J, Hampton AN, O'Doherty JP. Determining a role for ventromedial prefrontal cortex in encoding action-based value signals during reward-related decision making. *Cereb Cortex*. 2009;19(2):483-495. doi:10.1093/cercor/bhn098

52. Niv Y. Reinforcement learning in the brain. *J Math Psychol*. 2009;53(3):139-154. doi:10.1016/j.jmp.2008.12.005

53. Gueorguieva R, Krystal JH. Move Over ANOVA. *Arch Gen Psychiatry*. 2004;61(3):310. doi:10.1001/archpsyc.61.3.310

54. Gelman A, Hill J. *Data Analysis Using Regression and Multilevel/Hierarchical Models*.

Cambridge University Press; 2006.

55. Gallop R, Tasca GA. Multilevel modeling of longitudinal data for psychotherapy researchers: II. the complexities. *Psychother Res*. 2009;19(4-5):438-452. doi:10.1080/10503300902849475

eFigure 1. Association of overall performance with MDD diagnosis and symptom severity

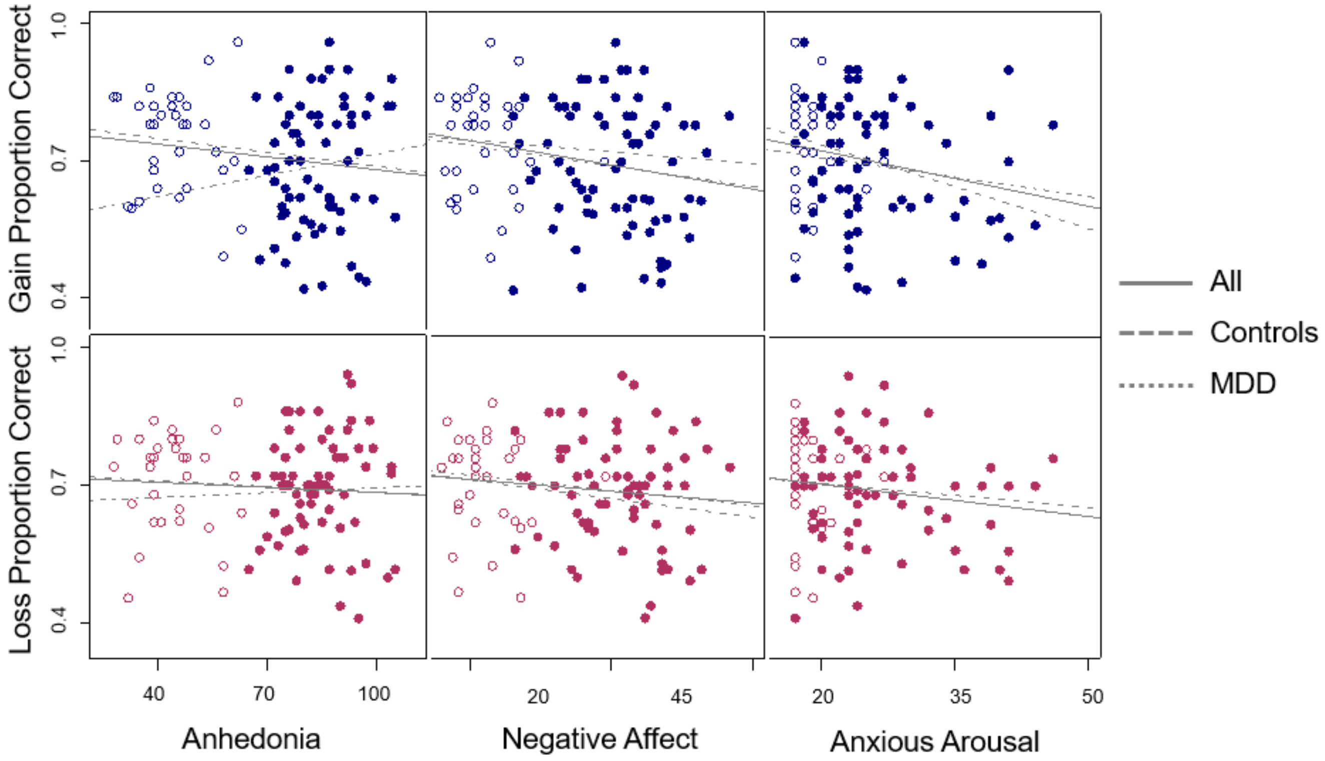

eFigure 1. Relationship of overall performance with symptom severity. Performance (proportion correct choices) is plotted versus symptom severity measures (anhedonia [MASQ anhedonia], negative affect [MASQ general distress], and anxious arousal [MASQ anxious arousal]). Control participants' data are indicated by open circles and participants with a depression diagnosis by filled circles. Top panels (navy) show gain learning performance while bottom panels (maroon) show loss learning performance. Regression lines for the relationship between performance and symptom severity are plotted for all participants (solid line), control participants only (dashed line), and participants with a depression diagnosis only (dotted line).

© 2021 Brown VM et al. *JAMA Psychiatry.*

eFigure 2. Model fit and parameter recovery

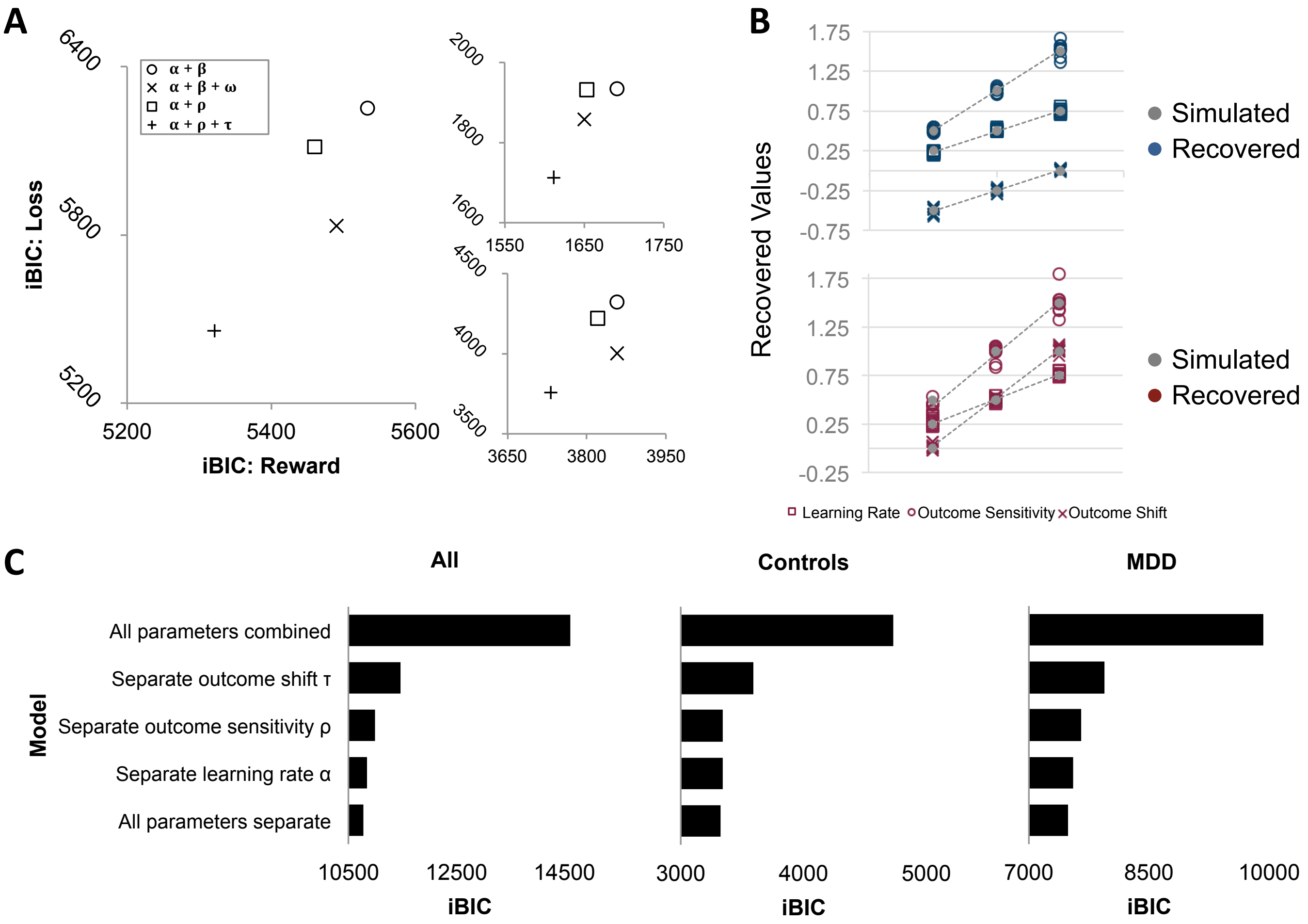

eFigure 2. Model fit and parameter recovery. **A)** The model with learning rate α, outcome sensitivity ρ, and outcome shift τ fits better across all participants (large left panel), control participants (top right panel), and participants with depression (bottom right panel) across reward (x axis) and loss (y axis) learning, relative to plausible alternative models of: learning rate α and inverse temperature β; learning rate α, inverse temperature β, and value-independent perseveration ω; and learning rate α and outcome sensitivity ρ. Values shown are integrated BIC (iBIC) values [77]. Smaller iBIC values indicate a better fitting model. **B)** Parameter values can be independently recovered from simulated data; 100 participants with mean parameter values at three different levels, determined based on the range of real participants' values, were simulated and recovered. Top panel shows reward parameters and bottom panel shows lossparameters, with simulated values indicated by gray dots connected by dotted gray lines, and recovered values indicated by navy (reward) or maroon (loss) symbols. Squares indicate recovered learning rate values, circles indicate recovered outcome sensitivity values, and crosses indicate recovered outcome shift values. **C)** Separating all parameters by valence (reward and loss) fits better across all participants, within control participants only, and within participants with depression only, relative to models combining one or all parameters across valence. Values shown are integrated BIC (iBIC) values.

© 2021 Brown VM et al. *JAMA Psychiatry.*

eFigure 3. Posterior distributions and individual means of parameters

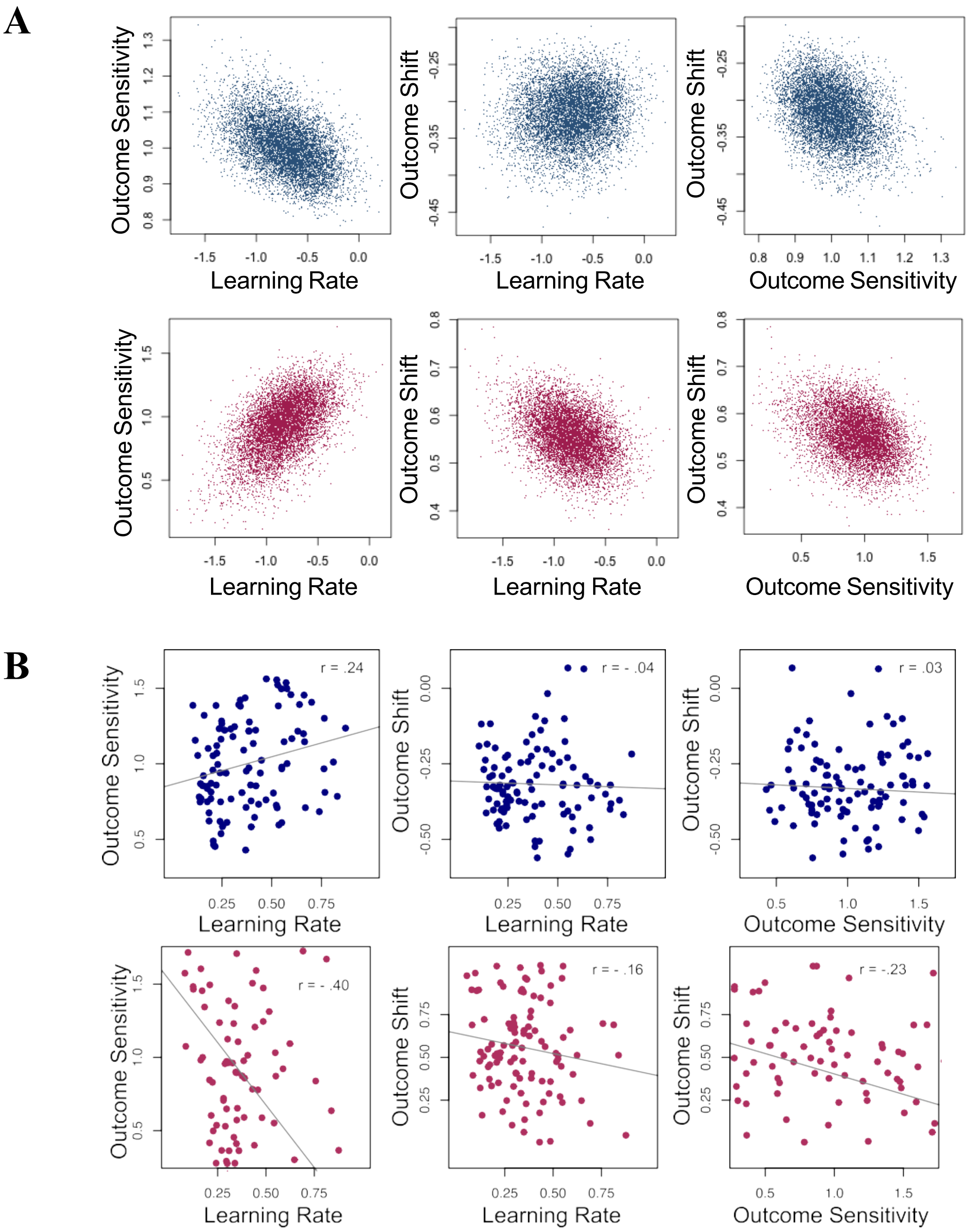

eFigure 3. Posterior distributions and individual means of parameters. A) Posterior distributions. Top panels (navy) indicate relationships among posterior distributions of reward parameters and bottom panels (maroon) of loss parameters. Each dot represents a sample from the posterior distribution of all participants included in baseline analyses during MCMC sampling (after discarding warm-up samples). **B)** Individual parameter means. Top panels (navy) indicate correlations of means of individual reward parameters and bottom panels (maroon) of individual loss parameters. Both sets of plots indicate a lack of collinearity among parameters.

© 2021 Brown VM et al. *JAMA Psychiatry.*

eFigure 4. Association of model parameters with model-agnostic summaries of behavior

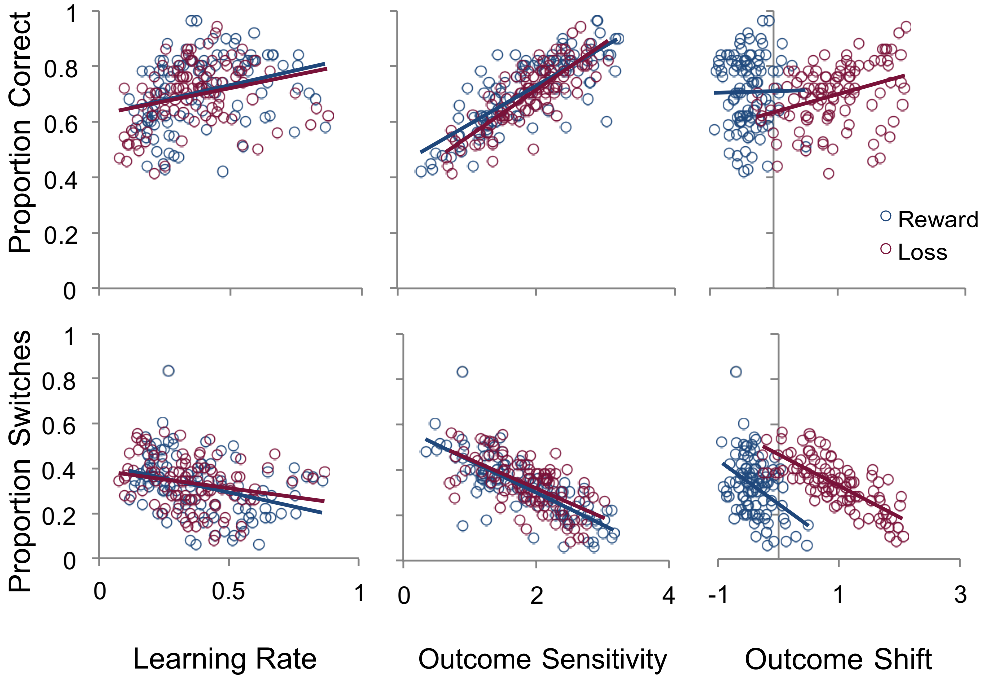

eFigure 4. Relationship of model parameters with model-agnostic summaries of behavior. Parameters of learning rate (left column), outcome sensitivity (middle column), and outcome shift (right column) show differentiable relationships with overall proportion of correct choices (top row) and overall proportion of switches (bottom row). Navy circles denote values from reward learning and maroon circles denote values from loss learning. Learning rate was moderately related to performance, with low learning rates, reflecting slower acquisition of contingencies, related to lower proportion of correct choices (gain: $r^2 = .097$; loss: $r^2 = .079$) and more switches (gain: $r^2 = .122$; loss: $r^2 = .056$). Outcome sensitivity changes the value of the more extreme outcome and, by extension, changes the value difference between options. This parameter was related to the ability to pick the stimulus more likely to lead to a better outcome (i.e., proportion correct choices; gain: $r^2 = .613$; loss: $r^2 = .690$) and to a reduced tendency to switch options (gain: $r^2 = .591$; loss: $r^2 = .404$), as would be expected with increased ability to differentiate among outcomes. Meanwhile, outcome shift changes all outcome values to be more positive or negative; this parameter was less related to the proportion of correct choices (gain: $r^2 = .0001$; loss: $r^2 = .117$) but was related to a reduced tendency to switch with higher valuation of outcomes (gain: $r^2 = .169$; loss: $r^2 = .560$), reflecting a tendency to switch away from any choice whenthe associated outcomes are perceived as more negative with a negative outcome shift value, and to stay with all choices when outcomes are viewed as positive with a more positive outcome shift value.

© 2021 Brown VM et al. *JAMA Psychiatry.*

eFigure 5. Association between anhedonia and reward learning parameters by diagnosis

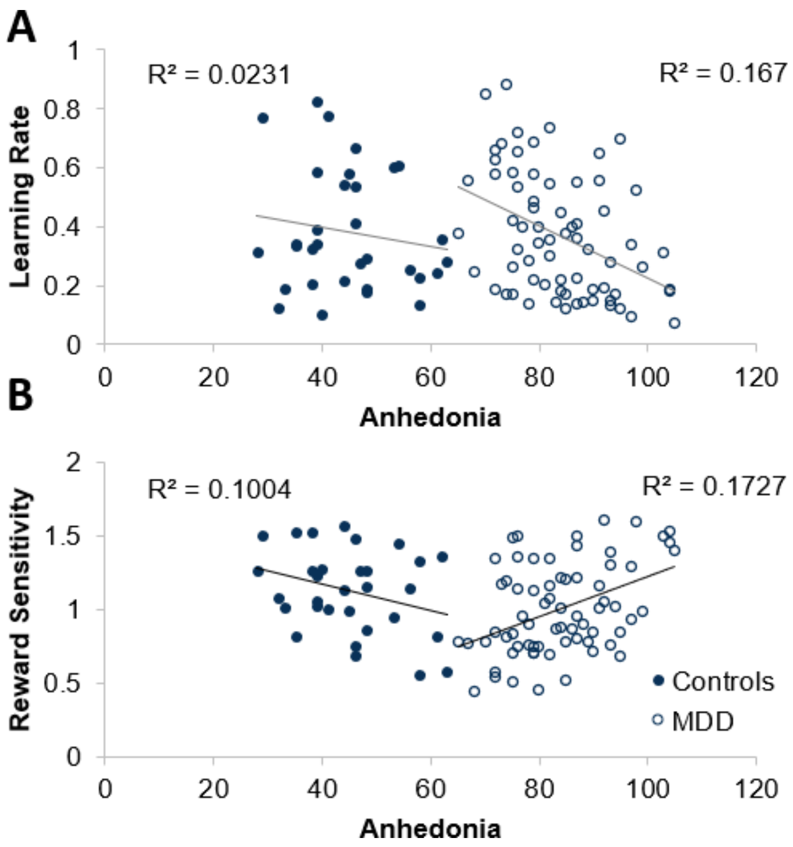

eFigure 5. Relationship between anhedonia and reward learning parameters by diagnosis. Dots represent individual parameter estimates per participant in models estimated separately in control (filled circles) and MDD (open circles) participants. X axis is anhedonia (MASQ anhedonia subscale) and Y axis is parameter estimates for learning rate (top) and reward sensitivity (bottom), with regression lines and R^2 values noting the relationship between anhedonia and parameter values for each diagnostic group. Note that partial pooling of individual estimates in the hierarchical estimation means that individual estimates should be used for illustration only and that R^2 values shown here may deviate somewhat from regression values reported in results.

© 2021 Brown VM et al. *JAMA Psychiatry.*

eFigure 6. Behavioral performance and neural reward signals by depression status and overall depression severity

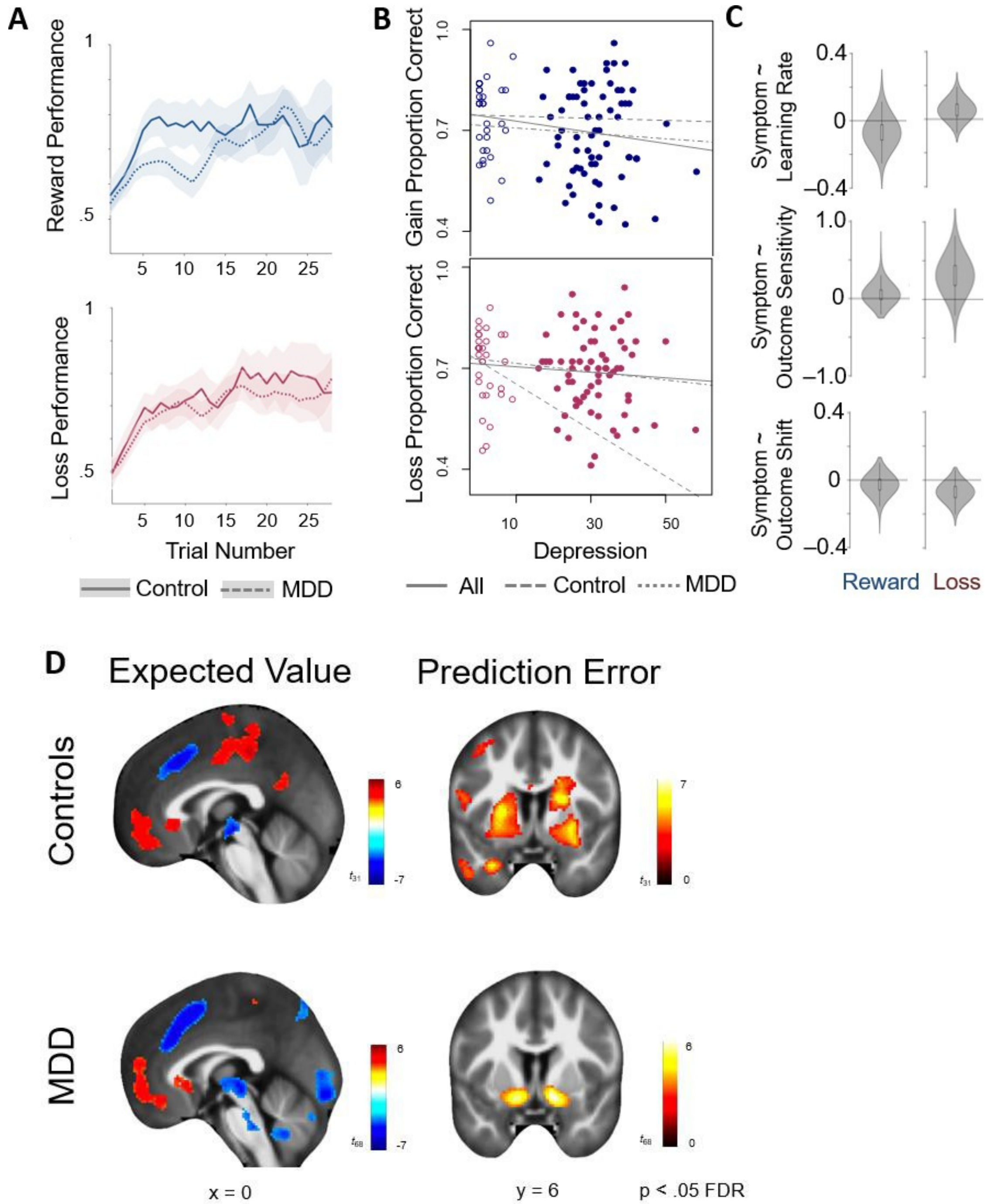

eFigure 6. Behavioral performance and neural reward signals by depression status and overall depression severity. **A**) Reward and loss learning performance by diagnosis. **B**) Relationship of overall performance with depression (BDI) by diagnosis. **C**) Depression (BDI) is unrelated to behavioral reward and loss learning parameters. **D**) Lack of differences in neural reward learning signals in depression. Left column is modulation of brain activity by the parametric modulator of expected value at time of stimulus onset and right column is prediction error at the time of outcome; top row is control

© 2021 Brown VM et al. *JAMA Psychiatry.*

participants and bottom row is participants with depression. Values are shown $p < .05$ whole brain FDR corrected ($p < .001$ cluster forming threshold). Significant clusters are reported in eTables 1-4.

© 2021 Brown VM et al. *JAMA Psychiatry.*

eFigure 7. Differences in processing of loss outcomes by negative affect

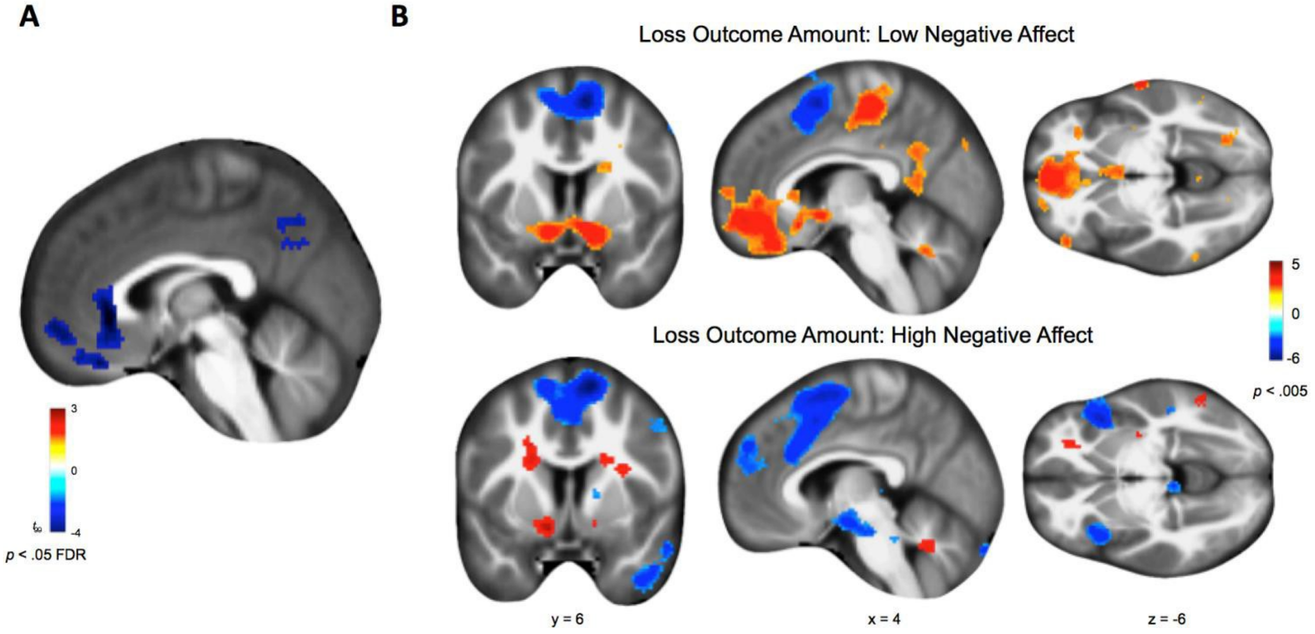

eFigure 7. Differences in processing of loss outcomes by negative affect. A) Significant whole-brain corrected differences by negative affect in modulation of brain activity with level of outcome (FDR p < .05, cluster forming threshold of p < .001; eTable 5). Group-level covariate of negative affect on a parametric modulator of outcome value at the time of outcome receipt. **B)** Processing of outcome value separated into low (top; eTable 6) and high (bottom; eTable 7) negative affect participants based on a median split, for the purposes of understanding the effect of negative affect shown in panel A. Note that both groups show robust responses that are modulated by outcome value (activation is significant p < .05 corrected & displayed at p < .005 uncorrected), but, reflecting the differences in signal by level of negative affect shown in A), these responses show a different spatial pattern and direction of activation in low and high negative affect participants. Specifically, participants with high negative affect showed activation negatively related to outcome value in dorsomedial prefrontal cortex and insula and no positive relationship between outcome value and vmPFC signal, indicating these participants engaged a different, more negatively-valenced network of brain areas rather than the positively-valenced regions of vmPFC and striatum engaged by low negative affect participants.

© 2021 Brown VM et al. *JAMA Psychiatry.*

eFigure 8. Stability of parameter estimates over time for control participants

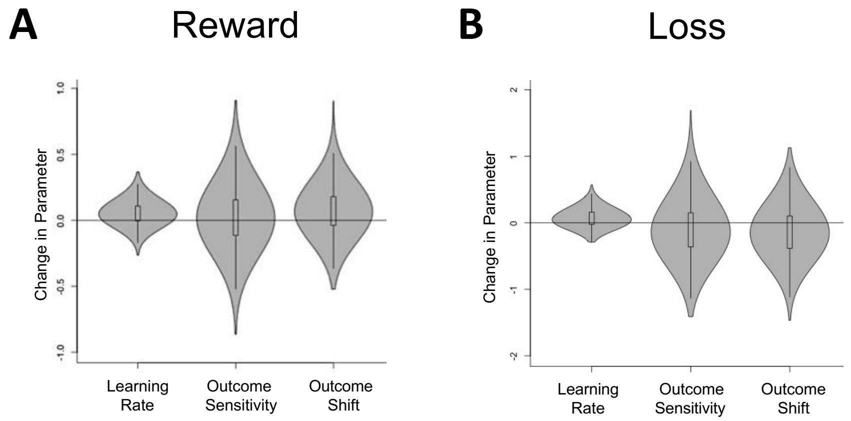

eFigure 8. Stability of parameter estimates over time for control participants. Left panel is reward parameters and right panel is loss parameters; violin plots indicate posterior densities of the within-subject change in each parameter from the first time point to the second, with a value of 0 representing no change in learning parameters.

eFigure 9. Parameter changes with time for participants with depression

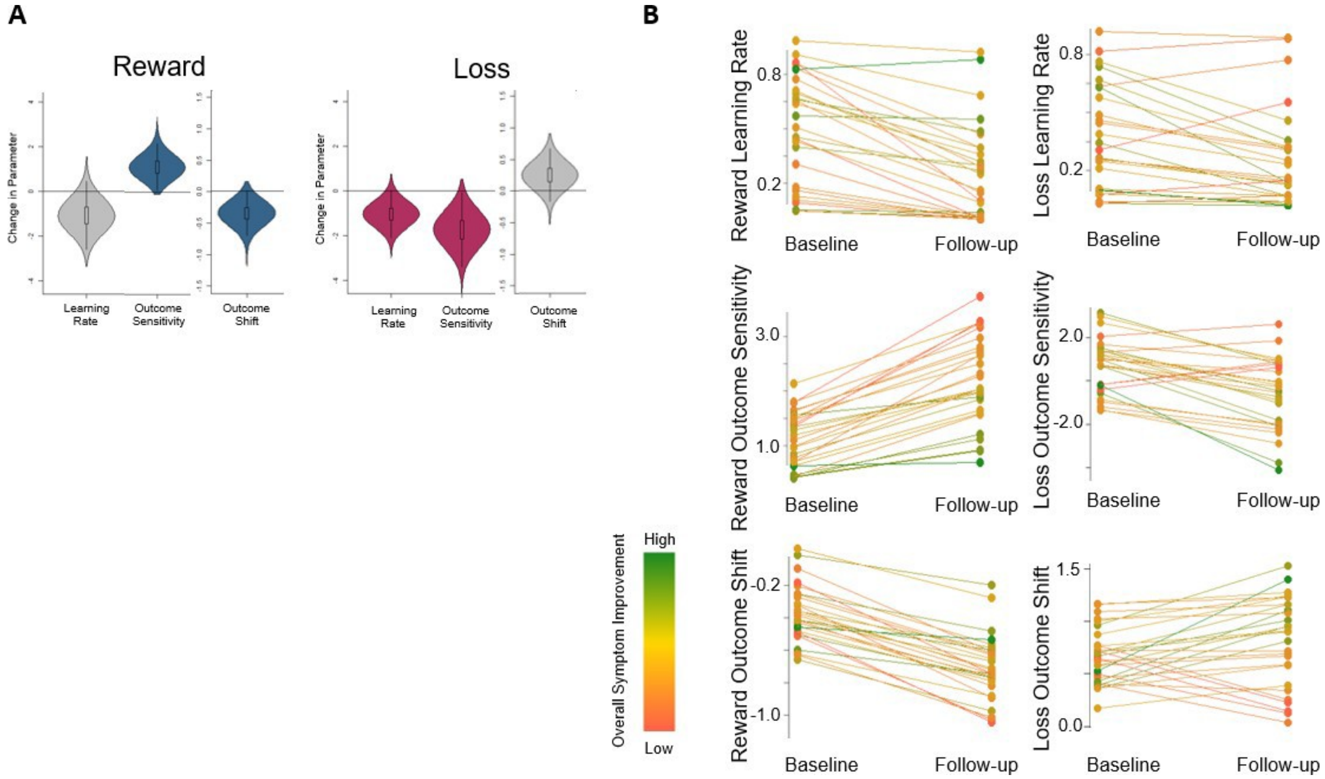

eFigure 9: Parameter changes with time for participants with depression. A) Left panel is reward parameters and right panel is loss parameters; violin plots indicate posterior densities of the within-subject change in each parameter, independent of symptom change, from the first time point to the second, with a value of 0 representing no change in learning parameters. Significant changes over time are represented by colored densities (navy for significant changes in reward learning parameters and maroon for significant changes in loss learning parameters). **B**) Changes in learning parameters from pre- to post-treatment for individual patients. Individual lines of parameter change are colored by degree of overall symptom improvement.

© 2021 Brown VM et al. *JAMA Psychiatry.*

eFigure 10. Diagram of flow of participants through study, including optional CBT portion

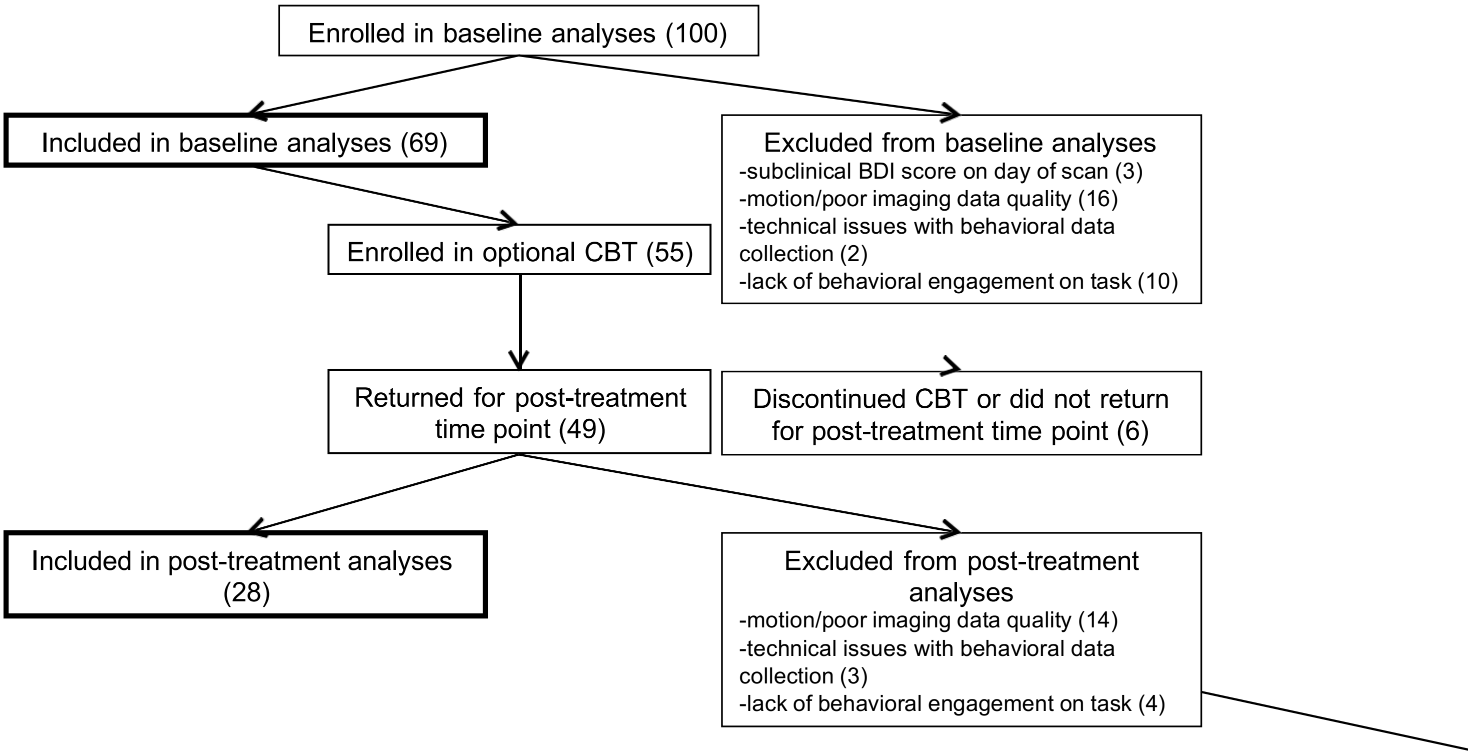

eFigure 10. Flow of participants with depression through study, including optional CBT portion. Bolded boxes on left side of diagram indicate final numbers for baseline analyses (top) and analyses of correlations with treatment (bottom). Note: this diagram does not include non-psychiatric control participants: N=32 included for baseline analyses and N=20 included in follow-up analyses matched to depressed participants' post-treatment time points.

© 2021 Brown VM et al. *JAMA Psychiatry.*

eFigure 11. Neural responses associated with symptom improvement

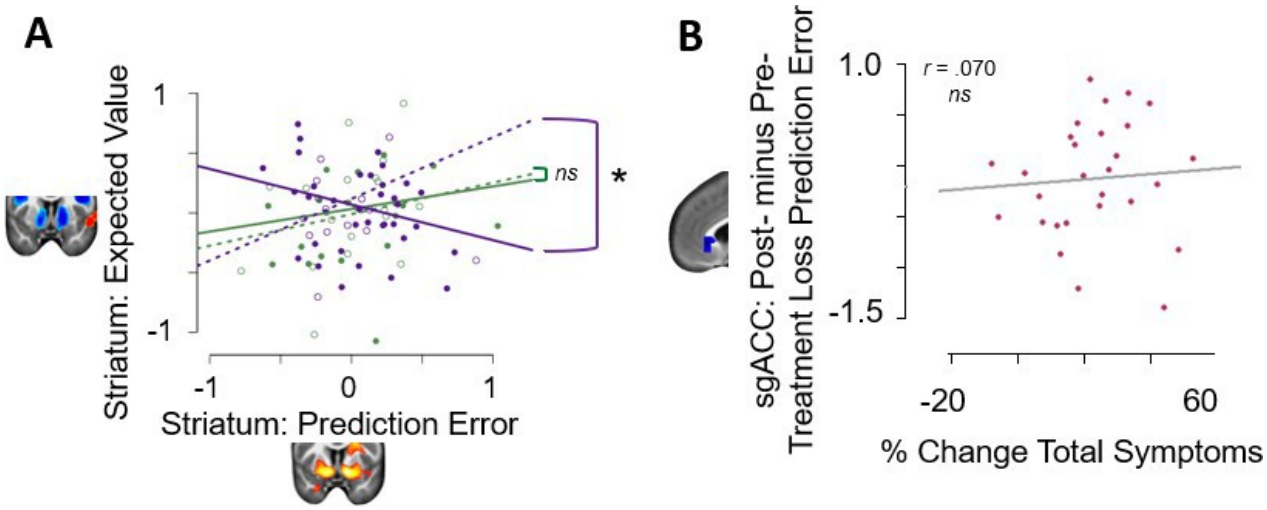

eFigure 11. Neural responses associated with symptom improvement. A) Reward learning: moderation of striatal expected value-prediction error relationship by pre-treatment anhedonia is significantly reduced post-treatment. Filled dots indicate values at baseline and open dots indicate follow-up values. Green dots indicate participants with low baseline anhedonia (non-depressed controls,assessed at matched timepoints to patients) and purple dots indicate participants with high baseline anhedonia. Values are right striatum region of interest activation to prediction error (x axis) and expected value (y axis). Solid lines represent regression lines pre-treatment and dotted lines represent regression lines post-treatment, with a significant change in slope for the high anhedonia group and no change for controls. **B)** Loss learning: change in sgACC signal pre- to post-treatment does not correlate with changes in symptoms. Maroon dots indicate patients' percent change in symptoms (total MASQ) versus post- minus pre-treatment sgACC region of interest activation to prediction error. Line is regression line showing lack of relationship.

© 2021 Brown VM et al. *JAMA Psychiatry.*

eFigure 12. Schematic depiction of effects of outcome sensitivity and outcome shift on valuation

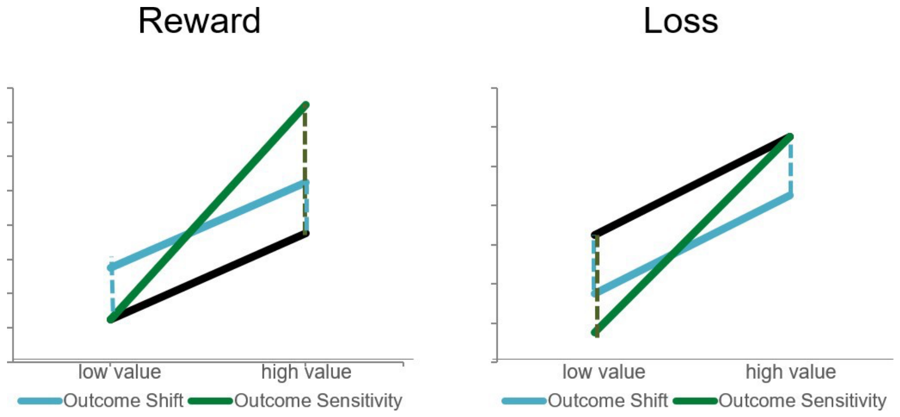

eFigure 12. Schematic depiction of effects of outcome sensitivity and outcome shift on valuation. The relationship between low and high values is illustrated with the black line and changes effected by each parameter are illustrated with colored lines. Outcome sensitivity (green) is multiplied on the more extreme value (high reward/low loss) and changes the slope of values. Outcome shift (light blue) is added to all values and changes the intercept of values**.**

© 2021 Brown VM et al. *JAMA Psychiatry.*

eTable 1. Reward prediction error, MDD group (n = 69)

Cluster Number	Region	Peak MNI Coordinate			Peak T Value	Cluster Size
1	Right ventral striatum	14	6	-12	6.04	2001
	Ventromedial prefrontal cortex	*-12*	*38*	*-10*	*4.74*	
2	Left ventral striatum	-16	6	-12	5.53	812
3	Left precuneus	-4	-50	32	5.3	848
4	Left superior parietal lobule	-32	-78	46	4.43	323
5	Left cerebellum	-14	-82	-24	3.94	213

© 2021 Brown VM et al. *JAMA Psychiatry.*

eTable 2. Reward expected value, MDD group (n = 69)

Cluster Number	Region	Peak MNI Coordinate			Peak T Value	Cluster Size
1	Right fusiform gyrus	32	-48	-18	-7.34	20497
	Left inferior parietal lobule	*-34*	*-62*	*44*	*-7*	
	Right occipital lobe	*36*	*-78*	*26*	*-6.87*	
2	Right middle frontal gyrus	48	48	18	-6.56	7339
	Right middle cingulate gyrus	*6*	*28*	*36*	*-6.5*	
3	Right calcarine sulcus	26	-38	18	6.33	608
4	Left calcarine sulcus	-26	-42	10	6.2	621
5	Right striatum	16	4	-4	-6.17	3403
	Left thalamus	*-6*	*-10*	*-4*	*-5.45*	
6	Left middle frontal gyrus	-52	24	34	-5.74	1335
7	Left insula	-30	20	4	-5.59	304
8	Right superior temporal gyrus	58	-32	14	5.34	1990
9	Left medial frontal gyrus	-8	60	16	5.17	1235
10	Left superior temporal gyrus	-54	-32	14	4.8	683
11	Right postcentral gyrus	16	-46	68	4.6	514
12	Right subgenual cingulate	6	24	-4	4.58	239
13	Left postcentral gyrus	-18	-48	68	4.38	160

© 2021 Brown VM et al. *JAMA Psychiatry.*

eTable 3. Reward prediction error, controls without depression (n = 32)

**Cluster Number**	**Region**	**Peak MNI Coordinate**			**Peak T Value**	**Cluster Size**
1	Left striatum	-24	0	-4	7.65	21994
	Right caudate	*20*	*4*	*16*	*6.57*	
2	Right cerebellum	20	-80	-28	7.31	8522
	Left middle temporal gyrus	*-60*	*-44*	*-10*	*6.94*	
	Left cerebellum	*-36*	*-72*	*-46*	*6.57*	
3	Left angular gyrus	-50	-68	26	5.68	5614
	Posterior cingulate gyrus	-2	-36	38	5.2	
4	Right middle frontal gyrus	28	38	46	5.19	893
5	Right middle temporal gyrus	58	-38	-12	4.26	213

© 2021 Brown VM et al. *JAMA Psychiatry.*

eTable 4. Reward expected value, controls without depression (n = 32)

Cluster Number	Region	Peak MNI Coordinate			Peak T Value	Cluster Size
1	Right inferior parietal lobule	42	-62	42	-7.44	2077
2	Right middle frontal gyrus	46	10	34	-6.94	4764
	Right middle cingulate gyrus	8	18	44	-6.58	
3	Midbrain	-8	-14	-12	-6.9	424
4	Left inferior frontal gyrus	-42	46	12	-6.29	745
5	Right precentral gyrus	20	-20	78	6.21	4684
6	Right insula	32	22	-8	-5.98	437
7	Right thalamus	16	-32	20	5.94	265
8	Left inferior parietal lobule	-36	-60	42	-5.62	1321
9	Left cerebellum	-38	-62	-50	-5.55	1909
10	Left thalamus	-18	-36	14	5.39	284
11	Right superior temporal gyrus	54	-10	-2	5.25	517
12	Right fusiform gyrus	32	-60	-10	-4.93	1154
13	Left inferior frontal gyrus	-46	2	30	-4.84	183
14	Subgenual anterior cingulate cortex	6	28	-4	4.78	170
15	Left insula	-32	18	-8	-4.55	284
16	Ventromedial prefrontal cortex	0	48	-20	4.46	582
17	Right striatum	16	4	-2	-4.4	151
18	Left precuneus	-8	-54	26	4.19	269

© 2021 Brown VM et al. *JAMA Psychiatry.*

eTable 5. Loss outcome value correlated with negative affect (MASQ Mixed Distress subscale; n = 101)

Cluster Number	Region	Peak MNI Coordinate			Peak T Value	Cluster Size
1	Left precuneus	-6	-64	44	-4.46	277
2	Subgenual anterior cingulate	4	32	-2	-4.39	976

© 2021 Brown VM et al. *JAMA Psychiatry.*

eTable 6. Loss outcome value, low negative affect participants (n = 52)

Cluster Number	Region	Peak MNI Coordinate			Peak T Value	Cluster Size
1	Left middle frontal gyrus	-20	32	44	6.5	848
2	Right superior frontal gyrus	8	8	60	-5.87	869
3	Ventromedial prefrontal cortex	4	54	-10	4.86	1287
4	Right supplementary motor area	4	-26	58	4.63	1357
5	Right ventral striatum	12	4	-14	4.61	190
6	Left precuneus	-12	-46	40	4.44	578
7	Right cerebellum	52	-72	-42	4.39	200

© 2021 Brown VM et al. *JAMA Psychiatry.*

eTable 7. Loss outcome value, high negative affect participants (n = 49)

Cluster Number	Region	Peak MNI Coordinate			Peak T Value	Cluster Size
1	Right supplementary motor area	12	6	68	-6.09	2417
2	Right insula	42	16	-6	-5.23	430
3	Right precentral gyrus	24	-10	36	5	618
4	Right inferior parietal lobe	40	-40	26	4.68	269
5	Left insula	-40	24	0	-4.49	283
6	Left postcentral gyrus	-34	-28	32	4.27	381

Note: results in all fMRI activation tables are based on whole brain correction at p < .05 cluster corrected with topological FDR using a p < .001 cluster forming threshold.

© 2021 Brown VM et al. *JAMA Psychiatry.*

eTable 8. Depression diagnosis, specifier, severity, medications, and comorbid diagnoses for participants

Primary Diagnosis	N	MDD Severity	N	MDD Specifier	N	Medications[1]	N	Comorbid Diagnoses[2]	N
None	32	N/A[3]	35	N/A[4]	40	None	78	Nicotine Dependence	10
MDD, single episode	13	mild	15	neither	26	Antidepressant only	10	Posttraumatic Stress Disorder	9
MDD, recurrent	47	moderate	39	atypical	12	Antianxiety only	5	Obsessive-Compulsive Disorder	4
						Sleep only	4	Panic Disorder	8
Dysthymia	3	severe	12	melancholic	23	Antidepressant + antianxiety	3	Social Phobia	7
Dysthymia + MDD, recurrent	2					Antidepressant + sleep	1	Agoraphobia	7
Dysthymia + MDD, single episode	1					Antidepressant + antianxiety +mood stabilizer	1	Anxiety Disorder NOS	1

[1]Psychotropic medications; categories based on [93]
[2]Ns are counted for each comorbid diagnosis separately. Participants with multiple comorbid diagnoses were: 2 participants with panic disorder + agoraphobia + social phobia; 1 participant with panic disorder + PTSD, 1 with panic disorder + agoraphobia, 1 panic disorder + nicotine dependence, 1 agoraphobia + PTSD, 1 agoraphobia + social phobia, 1 social phobia + nicotine dependence, 1 agoraphobia + PTSD + OCD, 1 social phobia + PTSD + nicotine dependence, 1 agoraphobia + nicotine dependence
[3]No diagnosis or dysthymia only
[4]No diagnosis, dysthymia only, or information not available

eTable 9. Exploratory follow-up analyses of associations between symptom change and reinforcement learning parameters in participants with depression who completed CBT

Valence	Symptom Cluster	Parameter	Regression β (mean)	Regression β (mean, standardized)	Lower Bound	Upper Bound
Reward	Anhedonia (Change)	**Learning Rate (Change)**	**0.143**	**3.532**	**0.047**	**0.264**
		**Outcome Sensitivity (Change)**	**-0.517**	**-4.167**	**-0.727**	**-0.322**
		Outcome Shift (Change)	0.033	0.406	-0.103	0.164
	Negative Affect (Change)	**Learning Rate (Change)**	**0.328**	**3.837**	**0.153**	**0.475**
		**Outcome Sensitivity (Change)**	**-0.541**	**-1.563**	**-1.080**	**-0.093**
		Outcome Shift (Change)	0.214	1.142	-0.098	0.451
	Arousal (Change)	**Learning Rate (Change)**	**0.118**	**1.972**	**0.031**	**0.224**
		**Outcome Sensitivity (Change)**	**-0.0367**	**-1.255**	**0.106**	**1.345**
		Outcome Shift (Change)	0.152	1.401	-0.027	0.327
Loss	Anhedonia (Change)	**Learning Rate (Change)**	**0.090**	**1.190**	**0.010**	**0.185**
		Outcome Sensitivity (Change)	1.188	1.929	-0.059	1.863
		Outcome Shift (Change)	-0.007	-0.066	-0.145	0.203
	Negative Affect (Change)	**Learning Rate (Change)**	**-0.042**	**-2.440**	**-0.077**	**-0.014**
		**Outcome Sensitivity (Change)**	**-1.281**	**-4.787**	**-1.732**	**-0.862**
		**Outcome Shift (Change)**	**0.419**	**5.086**	**0.290**	**0.559**
	Arousal (Change)	Learning Rate (Change)	-0.052	-1.242	-0.146	0.052
		Outcome Sensitivity (Change)	-0.510	-0.769	-1.440	0.729
		Outcome Shift (Change)	0.113	0.858	-0.125	0.305

Bolded values indicate significant relationships between changes in parameters and changes in symptoms following CBT. Change is measured as proportion improvement from pre- to post-CBT, so a positive relationship indicates greater increases in a parameter value in participants with greater symptom improvement.

© 2021 Brown VM et al. *JAMA Psychiatry.*
